# Discovery and characterization of differentially expressed soybean miRNAs and their targets during soybean mosaic virus infection unveils novel insight into Soybean-SMV interaction

**DOI:** 10.1186/s12864-022-08385-z

**Published:** 2022-03-02

**Authors:** Bowen Li, Adhimoolam Karthikeyan, Liqun Wang, Jinlong Yin, Tongtong Jin, Hui Liu, Kai Li, Junyi Gai, Haijian Zhi

**Affiliations:** 1 National Key Laboratory of Crop Genetics and Germplasm Enhancement, Key Laboratory of Biology and Genetic Improvement of Soybean (General, Ministry of Agriculture), National Center for Soybean Improvement, Nanjing Agricultural University, Nanjing, 210095 People’s Republic of China; 2 Subtropical Horticulture Research Institute, Jeju National University, Jeju, 63243 South Korea

**Keywords:** Soybean, Soybean mosaic virus, High-throughput sequencing, MicroRNAs, Target genes

## Abstract

**Background:**

Soybean mosaic virus (SMV) is one of the most devastating pathogens of soybean. MicroRNAs (miRNAs) are a class of non-coding RNAs (21–24 nucleotides) which are endogenously produced by the plant host as part of a general gene expression regulatory mechanisms, but also play roles in regulating plant defense against pathogens. However, miRNA-mediated plant response to SMV in soybean is not as well documented.

**Result:**

In this study, we analyzed 18 miRNA libraries, including three biological replicates from two soybean lines (Resistant and susceptible lines to SMV strain SC3 selected from the near-isogenic lines of Qihuang No. 1 × Nannong1138-2) after virus infection at three different time intervals (0 dpi, 7 dpi and 14 dpi). A total of 1,092 miRNAs, including 608 known miRNAs and 484 novel miRNAs were detected. Differential expression analyses identified the miRNAs profile changes during soybean-SMV interaction. Then, miRNAs potential target genes were predicted via data mining, and functional annotation was done by Gene Ontology (GO) analysis. The expression patterns of several miRNAs were validated by quantitative real-time PCR. We also validated the miRNA-target gene interaction by agrobacterium-mediated transient expression in *Nicotiana benthamiana*.

**Conclusion:**

We have identified a large number of miRNAs and their target genes and also functional annotations. We found that multiple miRNAs were differentially expressed in the two lines and targeted a series of NBS-LRR resistance genes. It is worth mentioning that many of these genes exist in the previous fine-mapping interval of the resistance gene locus. Our study provides additional information on soybean miRNAs and an insight into the role of miRNAs during SMV-infection in soybean.

**Supplementary Information:**

The online version contains supplementary material available at 10.1186/s12864-022-08385-z.

## Background

Soybean (*Glycine max* (L.) Merr.) is an important legume crop with numerous uses for food and industrial materials [1–3]. Soybean mosaic virus (SMV), a member of the genus *Potyvirus*, is a single-stranded sense RNA with about 9,600 nucleotides and encodes 11 putative proteins [4–6]. It is one of the most economically upsetting pathogens of soybean worldwide, and 50–80% yield losses due to SMV infection had been reported. This virus is predominantly found in many parts of the soybean-producing regions in China, and a total of 22 strains (designated as SC1-SC22) were identified nationwide [7, 8]. Considering the impact of SMV on soybean production, deciphering the molecular mechanisms of the interaction between soybean and SMV is of prime importance and is necessary for soybean improvement programs to increase the yield levels.

MicroRNAs (miRNAs) are a class of endogenous small non-coding RNAs that are 21–24 nucleotides (nt) long and have essential roles in regulating gene expression at the transcriptional and post-transcriptional level [9–13]. The miRNA encoding gene is initially transcribed by RNA polymerase II to produce a long primary-miRNA (pri-miRNA) molecule with a 5’ cap and 3’ poly-A tail. The original transcript is cleaved by RNAse III (Drosha and Dicer) in a single-stranded RNA precursor (pre-miRNA) with a hairpin structure of about 70–90 bases in size and then processed by Dicer to form mature miRNAs. Mature-miRNAs are assembled into the RNA-induced silencing complex (RISC) in the cell. In the RISC, base complementation guides the shearing of its target gene mRNA or inhibits its translation [9, 11, 14–16].

To date, thousands of miRNAs have been discovered in different plant species, and more are continuing to be discovered [17]. The role of miRNAs in regulating the expression of genes involved in various biological processes, including growth and development [18–20], phytohormone signaling [21, 22], immunity against pathogens and insect pests [23–25], and responses to environmental changes [26, 27], have been demonstrated. The majority of miRNAs have the ability to couple with several targets. Plant miRNAs and their putative target genes have been identified to be responsive to infection by viruses such as cucumber green mottle mosaic virus (CGMMV), mungbean yellow mosaic India virus (MYMIV), cowpea severe mosaic virus (CPSMV), sugarcane mosaic virus (SCMV) in cucumber, black gram, common bean, cowpea and maize [28–31].

With the support of high-throughput sequencing platforms and bioinformatics analysis, more than 700 miRNAs have been discovered in soybean. Through the analysis of four small RNA libraries, Li et al. [32] detected 101 miRNAs induced by soybean cyst nematode (SCN), indicating that miRNAs play a vital role in soybean response to SCN stress; Cui and Guo et al. [33, 34] also identified miR1510 and miR166, miR393, miR1507, miR2109, miR3533 in soybean in response to *Phytophthora sojae* infection.

However, there are few studies conducted to known about miRNAs involvement in SMV-soybean interactions. For instance, Yin et al. [35] found that miR160, miR393, and miR1510 were involved in plant resistance to SMV infection; Chen et al. [36, 37] performed small RNA (sRNA)-sequencing, degradome-sequencing and as well as a genome-wide transcriptome analysis to identify many miRNAs that responded to the infection of different SMV strains. Among them, the increase in the expression level of miR168 lead to a serious inhibition of the target AGO1 mRNA; Bao et al. [38] found that up-regulated miRNAs, miR168a, miR403a, miR162b and miR1515a may regulate the expression of AGO1, AGO2, DCL1 and DCL2 by microarray analysis.

In the present study, we identified a set of miRNAs responsive to SMV infection in resistant and susceptible near-isogenic lines (NILs) from the cross between Qihuang No.1 and Nannong1138-2 at different time intervals and provided a synopsis of the soybean miRNA and SMV interaction system. This information adds toward a better understanding of the role of miRNAs during SMV-infection in soybean.

## Results

### Post inoculation phenotype in resistant and susceptible lines

**Fig. 1:**
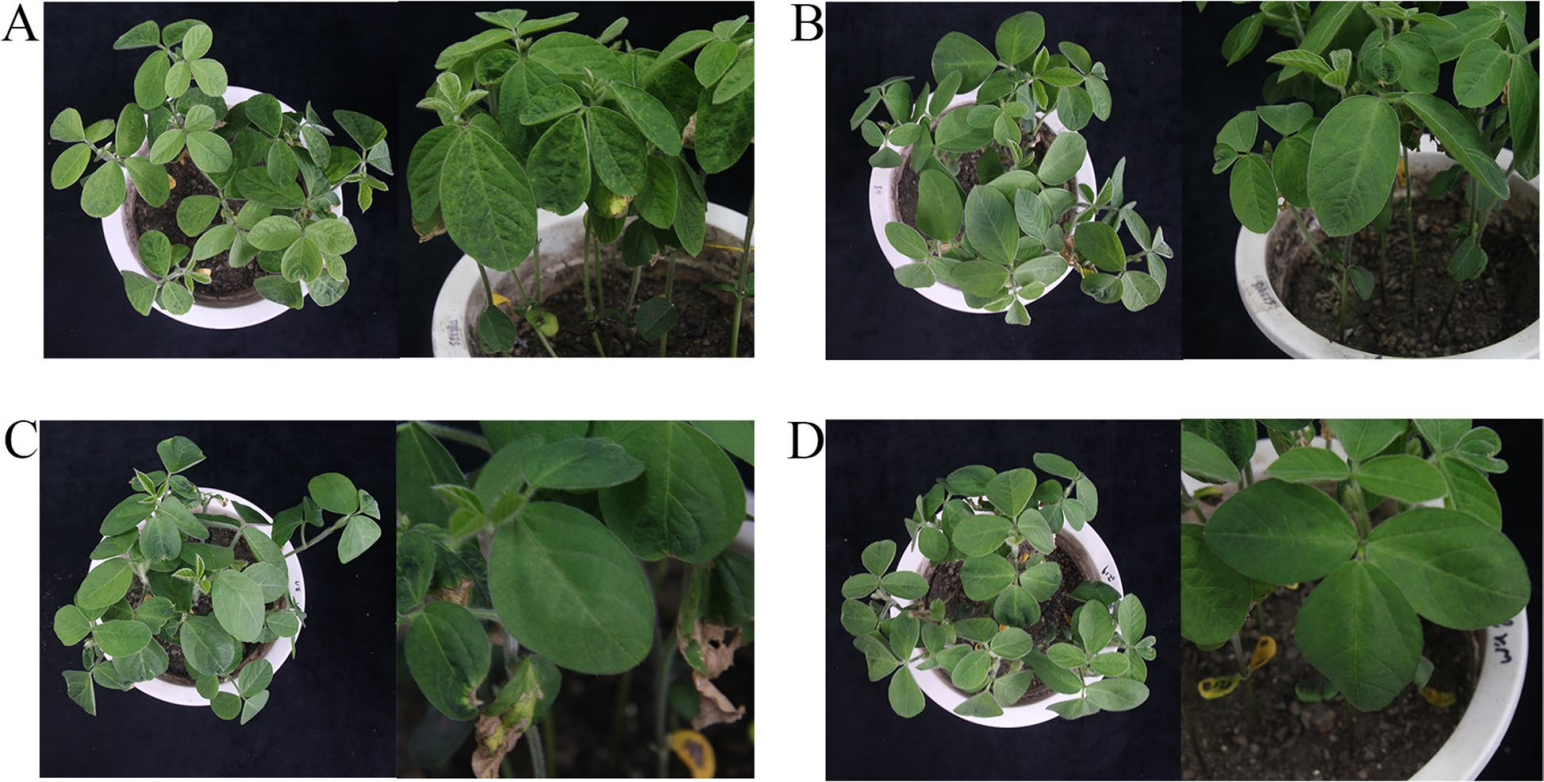
Phenotypic characteristics of resistant and susceptible lines inoculated with SC3 and PBS. **A**-**B** Leaves growth of susceptible line inoculated with SMV and PBS at 7 dpi, respectively. **C**-**D** Leaves growth of resistant line inoculated with SMV and PBS at 7 dpi, respectively. **A**, **C** were the phenotypes after inoculation with SMV, **B**, **D** were the phenotypes after inoculation with PBS

**Fig. 2:**
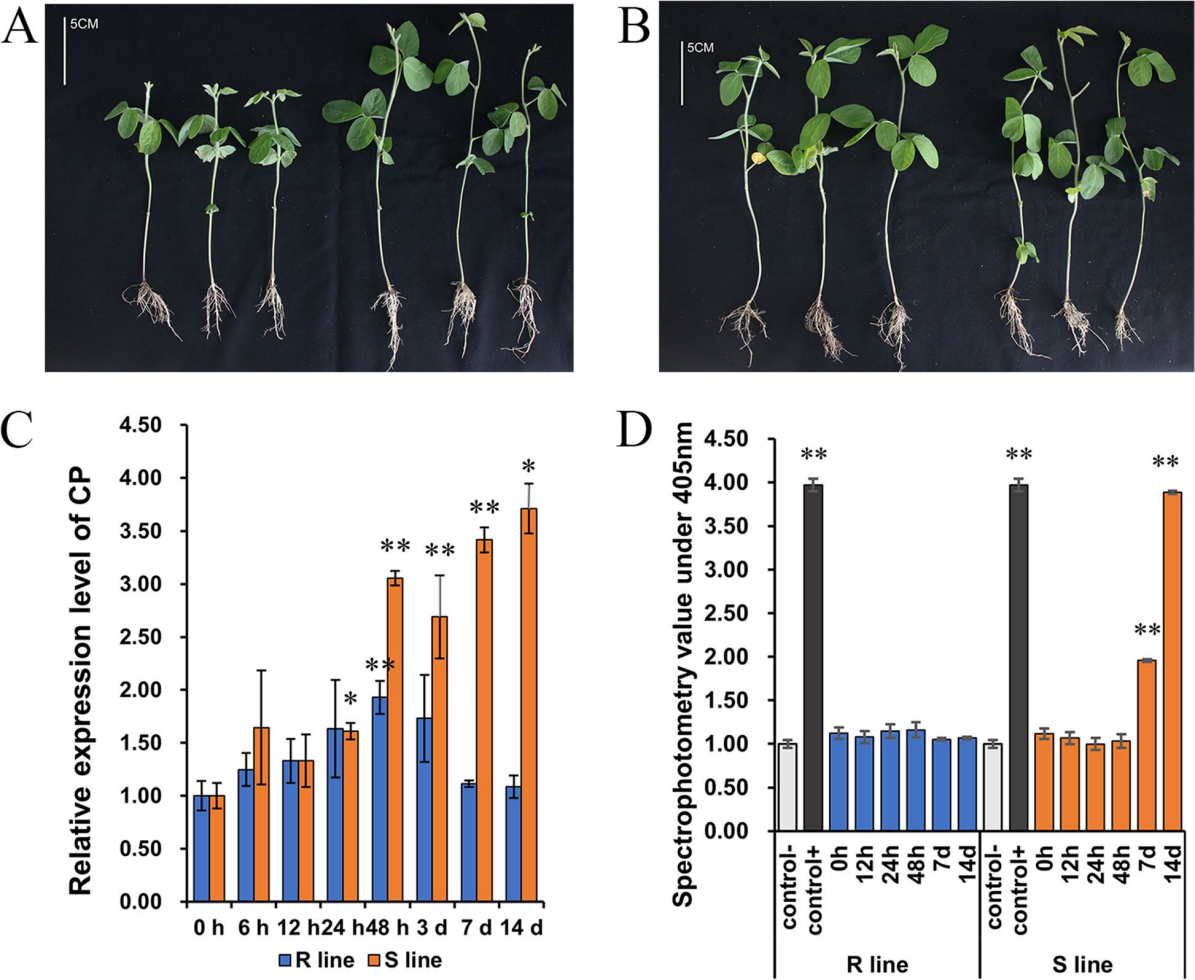
Phenotypic characteristics of plant height and detection of virus content after SC3 inoculation. **A** Comparison of plant height of susceptible line inoculation with SMV (3 on the left) and PBS (3 on the right) at 14 dpi. **B** Comparison of plant height of resistant line inoculation with SMV (3 on the left) and PBS (3 on the right) at 14 dpi. **C** qRT-PCR detection of the variation trend of viral content in the two lines after inoculation with SMV (for CP gene). Three biological repeats were set and the reference gene was Tubulin. The expression level of CP gene was calculated by 2^−ΔΔCT^. **D** DAS-ELISA detection of the viral content after SMV inoculation in two lines (for CP protein). Three biological replicates were set, control- was non-inoculated leaves of Nannong1138-2, and control + was infected leaves of Nannong1138-2 after inoculation with SC3

The resistant and susceptible lines were inoculated with SMV strain SC3 and phosphate buffer solution (PBS) with brushes, respectively, and their phenotypes were studied. As expected, SMV symptoms were not visible in mock-inoculated resistant and susceptible lines. The susceptible line resulted in yellow-green mosaic and slightly curling symptoms on the leaves. The onset of symptoms was observed 7 dpi onwards, and the mosaic and severe curling symptoms were at 14 dpi. In contrast, no visual disease symptoms were observed in the resistant line (Fig. 1). The plant height of the susceptible line was significantly lower than that of the control, while there was no significant difference seen in the resistant line and its control (Fig. 2A-B). The SMV-CP transcripts were quantified by qRT-PCR analysis. Resistant line showed low accumulation of the SMV-CP transcripts at 7 and 14 dpi compared to that of the susceptible line. SMV inoculation in the susceptible line resulted in a rise in an SMV-CP transcripts accumulation from 7 dpi onwards, which explains the phenotype changes observed. Further, this result was confirmed by DAS-ELISA analysis (Fig. 2C-D). These results indicated that the virus can gradually spread to the upper leaves in the susceptible line and can inhibit the normal growth of the plant, while the infection of the virus in the resistant line is blocked, allowing the plant to grow normally.

### Summary of small RNA libraries data sets

This study constructed 18 small RNA libraries from SMV-resistant and -susceptible lines at 0 dpi, 7 dpi and 14 dpi of SMV and generated 231.41 million raw reads ranging from 10.46 to 18.73 million reads each library. After removing the low-quality reads, a total of 225.42 million clean reads were obtained. Each sample was not less than 10.26 million clean reads, and the Q30 value of all samples was > 95% (Table 1). Next, we filtered out sRNAs such as ribosomal RNA (rRNA), transfer RNA (tRNA), small nuclear RNA (snRNA), small nucleolar RNA (snoRNA), and other noncoding RNAs (ncRNAs) and repetitive sequences in the clean reads to obtain unannotated reads containing miRNAs. Among all the reads in each sample, the total amount of rRNA and unannotated reads accounts for about 95% or more. Notably, small cytoplasmic RNAs (scRNAs) were not detected in any samples (Additional file 1: Figure S1). After comparing unannotated reads with the soybean reference genome (Wm82.a2. v1), it was found that the ratio of the sequence in the alignment ranges from 27.83 to 53.80%, most of which are around 40%. The number of clean reads compared to the positive chain for each sample is higher than the number of clean reads compared to the negative chain (Additional file 2: Table S1, Additional file 3: Figure S2).

**Table 1:** Summary of the sequencing data

Samples	Raw reads	Low quality	Containing 'N'reads	Length < 18	Length > 30		Clean reads	Q30(%)
R-0–1	10,943,860	0	0	160,919		0	10,782,941	96.05
R-0–2	10,462,389	0	0	199,240		0	10,263,149	95.30
R-0–3	11,474,337	0	0	313,220		0	11,161,117	96.78
R-7–1	14,104,702	0	0	526,839		0	13,577,863	96.51
R-7–2	12,147,715	0	0	531,193		0	11,616,522	96.40
R-7–3	11,769,498	0	0	311,735		0	11,457,763	95.94
R-14–1	11,115,807	0	0	333,049		0	10,782,758	96.60
R-14–2	11,142,894	0	0	430,347		0	10,712,547	96.47
R-14–3	11,179,221	0	0	185,087		0	10,994,134	95.78
S-0–1	11,060,909	0	0	372,772		0	10,688,137	95.52
S-0–2	12,724,182	0	0	415,931		0	12,308,251	95.92
S-0–3	18,737,861	0	0	471,647		0	18,266,214	96.04
S-7–1	13,915,992	0	0	318,714		0	13,597,278	95.92
S-7–2	13,027,907	0	0	178,403		0	12,849,504	95.95
S-7–3	11,647,746	0	0	191,013		0	11,456,733	95.95
S-14–1	15,553,512	0	0	246,334		0	15,307,178	96.16
S-14–2	17,450,155	0	0	410,914		0	17,039,241	96.32
S-14–3	12,954,807	0	0	400,776		0	12,554,031	96.47

Note: R-0–1, R-0–2, R-0–3 are samples taken from resistant line inoculated with SC3 at 0 dpi; S-0–1, S-0–2, S-0–3 are samples taken from susceptible line inoculated with SC3 at 0 dpi; R-7–1, R-7–2, R-7–3 are samples taken from resistant line inoculated with SC3 at 7 dpi;S-7–1, S-7–2, S-7–3 are samples taken from susceptible line inoculated with SC3 at 7 dpi; R-14–1, R-14–2, R-14–3 are samples taken from resistant line inoculated with SC3 at 14 dpi;S-14–1, S-14–2, S-14–3 are samples taken from susceptible line inoculated with SC3 at 14 dpi

### Identification of known and novel miRNA

**Fig. 3:**
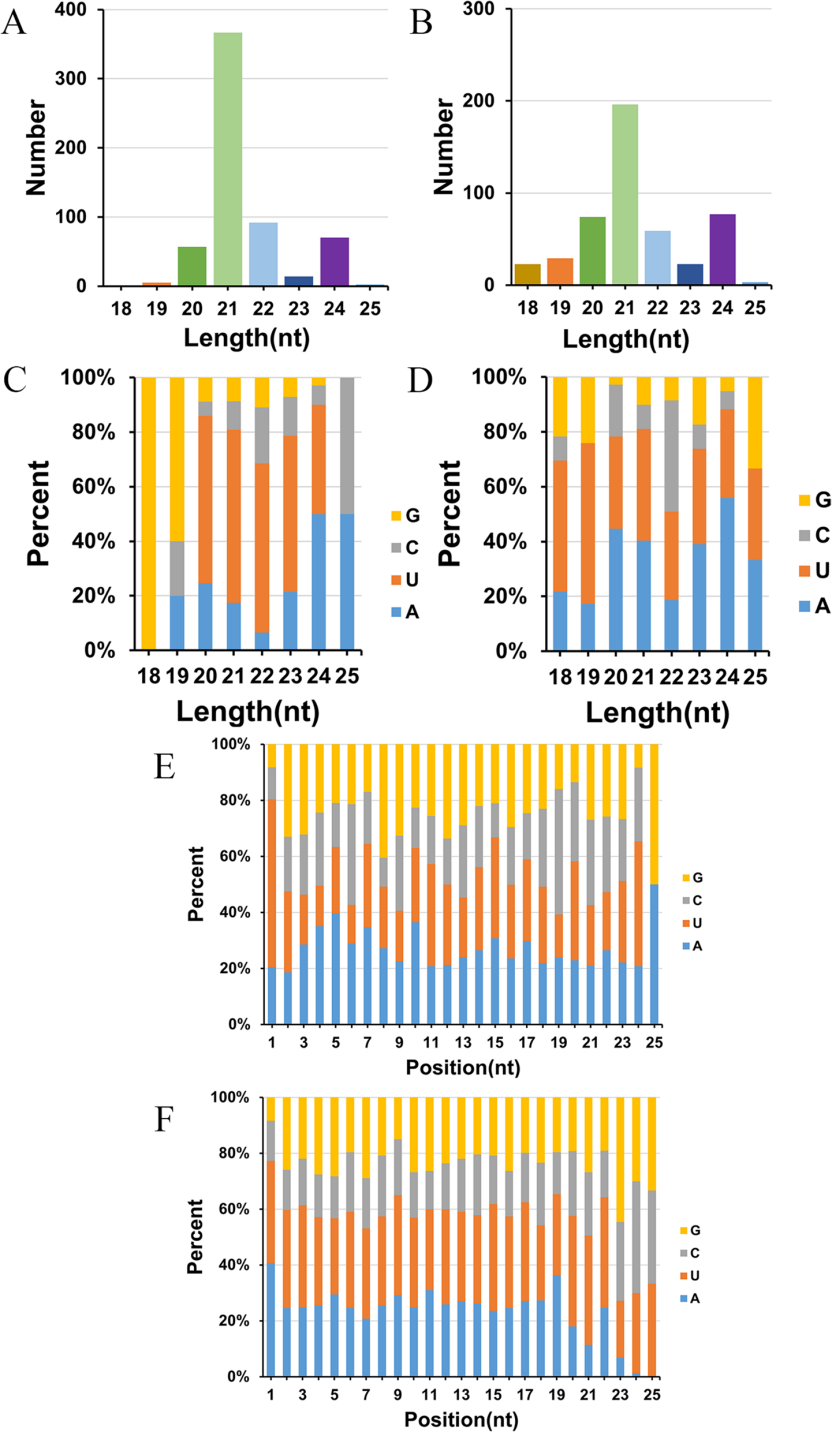
Characterization of the miRNAs. **A, C, E** The length distribution, the base preference of the first nucleotide, and the base preference of each position of all known miRNAs. **B, D, F** The length distribution, the base preference of the first nucleotide, and the base preference of each position of all novel miRNAs

A total of 1,092 miRNAs, including 608 known miRNAs and 484 novel miRNAs, were detected from all the libraries [39]. For known miRNAs, the number of miRNAs with a 21 nt length was the largest, followed by 22 nt and 24 nt. The proportion of miRNAs with a 20–23 nt length in which the first base of the 5’ end was U exceeds 50% (Fig. 3A, C, E). For the novel miRNAs, the number of miRNAs with a length of 21 nt was the largest, followed by 24 nt and 20 nt. The preference for U at the 5’end of these novel miRNAs was weaker than that of known miRNAs (Fig. 3B, D, F). Excluding miRNAs that still do not belong to miRNA families at present, for the remaining known miRNAs, 509 miRNAs belong to 108 miRNA families respectively. Of these, the MiR166 family having the highest number of miRNAs (18), followed by MiR169_2 (13), MiR159 (11), and MiR482 (11). At the novel miRNA level, 196 miRNAs in 80 families, of these, the MiR4352 family had the largest number of miRNAs (17), followed by MiR4378 (12) and MiR4374 (11) (Additional file 4: Table S2).

### Differentially expressed miRNAs after SMV infection

**Fig. 4:**
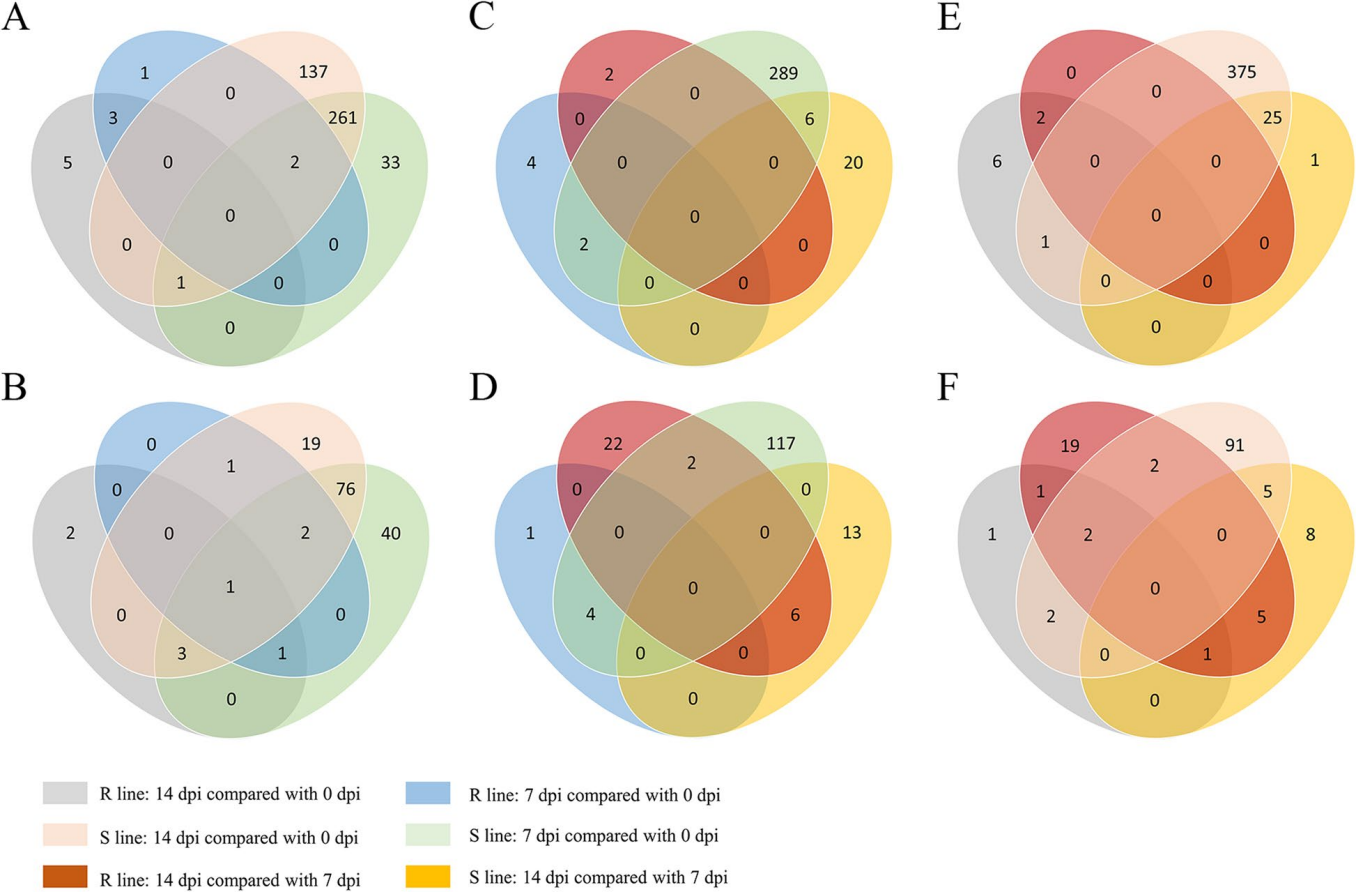
Comparative analysis of Wayne diagrams between different samples. **A**, **C**, **E** Up-regulation analysis between different groups. **B**, **D**, **F** Down-regulation analysis between different groups

To identify the differentially expressed miRNAs upon SMV infection, we analyzed the miRNAs expression by volcano plot. It revealed that the number of differentially expressed miRNAs in the susceptible line at different time points after inoculation with SMV was higher than that in the resistant line (Additional file 5: Figure S3). By comparing the two lines 0 dpi with 7 dpi with the Wayne diagram, we found that 4 miRNAs were up-regulated while 1 miRNA was down-regulated in the resistant line. On the other hand, 295 and 119 miRNAs were up- and down-regulated, respectively, in the susceptible line. Of these, two up-regulated miRNAs (gma-miR5761b, novel-miR-173) and four down-regulated miRNAs (gma-miR5037c, gma-miR5371-3p, gma-miR5371-5p, novel-miR-297) were common in both lines (Fig. 4A-F). Next, we compared the two lines 0 dpi with 14 dpi. It revealed that 8 miRNAs were up-regulated in the resistant line while 3 were down-regulated. In the susceptible line, 400 miRNAs were up-regulated, and 98 were down-regulated. One up-regulated miRNA (gma-miR391-5p) and four down-regulated (gma-miR5371-5p novel-miR-101, novel-miR-224, novel-miR-317) were common in both lines (Fig. 4A-F). Collectively, these results indicated that different miRNAs may be involved in the process of virus-plant interaction by responding to SMV infection at different stages of the resistant and susceptible lines.

Notably, among the 295 miRNAs up-regulated at 0 dpi to 7 dpi in the susceptible line, 7 miRNAs including novel-miR-50, novel-miR-70, novel-miR-449, novel-miR-466; gma-miR2118a-3p, gma-miR5041-5p, and gma-miR10440 were targeting the NBS-LRR genes. Moreover, among the 400 miRNAs up-regulated from 0 to 14 dpi in the susceptible line, 7 miRNAs were targeting NBS-LRR genes, 6 of which are the same as before, and the remaining one is gma-miR390d (Table 2), indicating that the up-regulation of miRNAs targeting NBS-LRR resistance genes in the susceptible line may lead to the down-regulation of corresponding target genes, which may potentially affect the disease resistance of plants.

**Table 2:** Summary of the miRNAs targeting NBS-LRR genes

miRNA	miRNA Seq	Target gene	R-0-1_R-0-2_R-0–3 Vs R-7-1_R-7-2_R-7–3	S-0-1_S-0-2_S-0–3 Vs S-7-1_S-7-2_S-7–3	R-0-1_R-0-2_R-0–3 Vs R-14-1_R-14-2_R-14–3	S-0-1_S-0-2_S-0–3 vs S-14-1_S-14-2_S-14–3
novel-miR-49	GAGAUUGGAGCAAUCAGAAUUUGUG	Glyma.18G287100.Wm82.a2.v1	down	normal	normal	normal
novel-miR-50	AAAUAAGAAGGAAUAAUGAA	Glyma.16G118600.Wm82.a2.v1	–	up	–	up
novel-miR-70	UGCGAGUGUCUUCGCCUCUG	Glyma.02G023800.Wm82.a2.v1	normal	up	normal	up
novel-miR-449	AAGAGGCUUGUAGAAGAAGUC	Glyma.15G168500.Wm82.a2.v1	normal	up	normal	up
novel-miR-466	AGCAUCUUAAAACACUUGAAU	Glyma.16G118600.Wm82.a2.v1	normal	up	–	up
gma-miR10440	UUGGGACAAUACUUUAGAUAU	Glyma.13G184800.Wm82.a2.v1	normal	up	normal	up
gma-miR10440	UUGGGACAAUACUUUAGAUAU	Glyma.13G188300.Wm82.a2.v1	normal	up	normal	up
gma-miR10440	UUGGGACAAUACUUUAGAUAU	Glyma.13G190400.Wm82.a2.v1	normal	up	normal	up
gma-miR10440	UUGGGACAAUACUUUAGAUAU	Glyma.13G190800.Wm82.a2.v1	normal	up	normal	up
gma-miR10440	UUGGGACAAUACUUUAGAUAU	Glyma.13G193300.Wm82.a2.v1	normal	up	normal	up
gma-miR1507a	UCUCAUUCCAUACAUCGUCUGA	Glyma.04G137800.Wm82.a2.v1	normal	down	normal	normal
gma-miR2118a-3p	UUGCCGAUUCCACCCAUUCCU	Glyma.13G184800.Wm82.a2.v1	normal	up	normal	up
gma-miR2118a-3p	UUGCCGAUUCCACCCAUUCCU	Glyma.13G187900.Wm82.a2.v1	normal	up	normal	up
gma-miR2118a-3p	UUGCCGAUUCCACCCAUUCCU	Glyma.13G188300.Wm82.a2.v1	normal	up	normal	up
gma-miR390d	AAGCUCAGGAGGGAUAGCACC	Glyma.20G100500.Wm82.a2.v1	normal	normal	normal	up
gma-miR5041-5p	UUUCAUCUUCAACUUGCUCAA	Glyma.13G190300.Wm82.a2.v1	normal	up	normal	normal
gma-miR5041-5p	UUUCAUCUUCAACUUGCUCAA	Glyma.13G190400.Wm82.a2.v1	normal	up	normal	normal
gma-miR5041-5p	UUUCAUCUUCAACUUGCUCAA	Glyma.13G193100.Wm82.a2.v1	normal	up	normal	normal
gma-miR5041-5p	UUUCAUCUUCAACUUGCUCAA	Glyma.13G193300.Wm82.a2.v1	normal	up	normal	normal

Note: miRNA: Differentially expressed miRNAs targeting NBS-LRR genes

miRNA Seq: The corresponding miRNA sequences

Target gene: NBS-LRR genes predicted by corresponding miRNAs

R-0–1, R-0–2, R-0–3 vs R-7–1, R-7–2, R-7–3: The variation trend of corresponding miRNAs from 7 to 0 dpi in resistant line

S-0–1, S-0–2, S-0–3 vs S-7–1, S-7–2, S-7–3: The variation trend of corresponding miRNAs from 7 to 0 dpi in susceptible line

R-0–1, R-0–2, R-0–3 vs R-14–1, R-14–2, R-14–3: The variation trend of corresponding miRNAs from 14 to 0 dpi in resistant line

S-0–1, S-0–2, S-0–3 vs S-14–1, S-14–2, S-14–3: The variation trend of corresponding miRNAs from 14 to 0 dpi in susceptible line

### Target gene prediction and annotation of differentially expressed miRNAs

**Fig. 5:**
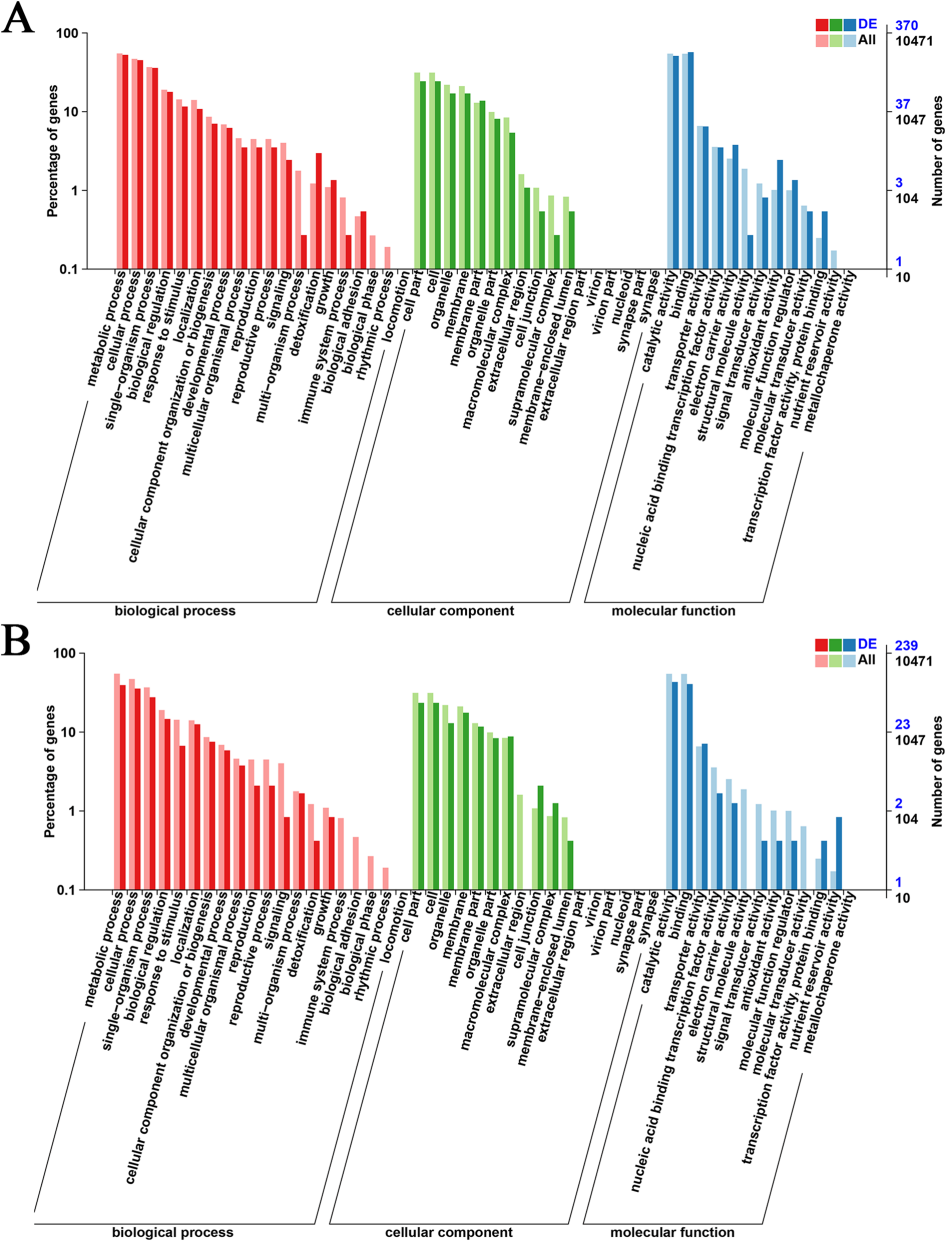
Gene ontology (GO) classification analysis of target genes of the differentially expressed miRNAs. **A** GO annotation of the target genes of differentially expressed miRNAs for 7 dpi compared with 0 dpi in resistant line. **B** GO annotation of target genes of differentially expressed miRNAs for 14 dpi compared with 0 dpi in resistant line. The x-axis represents three GO categories, the right y-axis represents the number of target genes in the category, and the left y-axis represents the percentage of target genes annotated with a specific term under the main category

The number of target genes annotated by differentially expressed miRNAs between different samples were analysed, and it was found that the minimum was 373 target genes at 0 dpi between the resistant and susceptible lines, and the maximum was 15,522 target genes in susceptible line from 0 to 14 dpi (Additional file 6: Table S3). The Gene Ontology (GO) analysis showed that: (1) From 0 to 7 dpi, the metabolic process, cell part, and binding were the most enriched terms in both lines under the biological process, cellular component, and molecular function categories (Fig. 5A, Additional file 7: Figure S4A). (2) From 0 to 14 dpi, the metabolic process cell and catalytic activity were the most enriched terms in the resistant line, respectively, while metabolic process cell, cell part binding, and catalytic activity were the most enriched terms in the susceptible line (Fig. 5B, Additional file 7: Figure S4B). It suggested that genes associated with different pathways in the two lines were likely to contribute to plants' resistance and susceptibility.

**Fig. 6:**
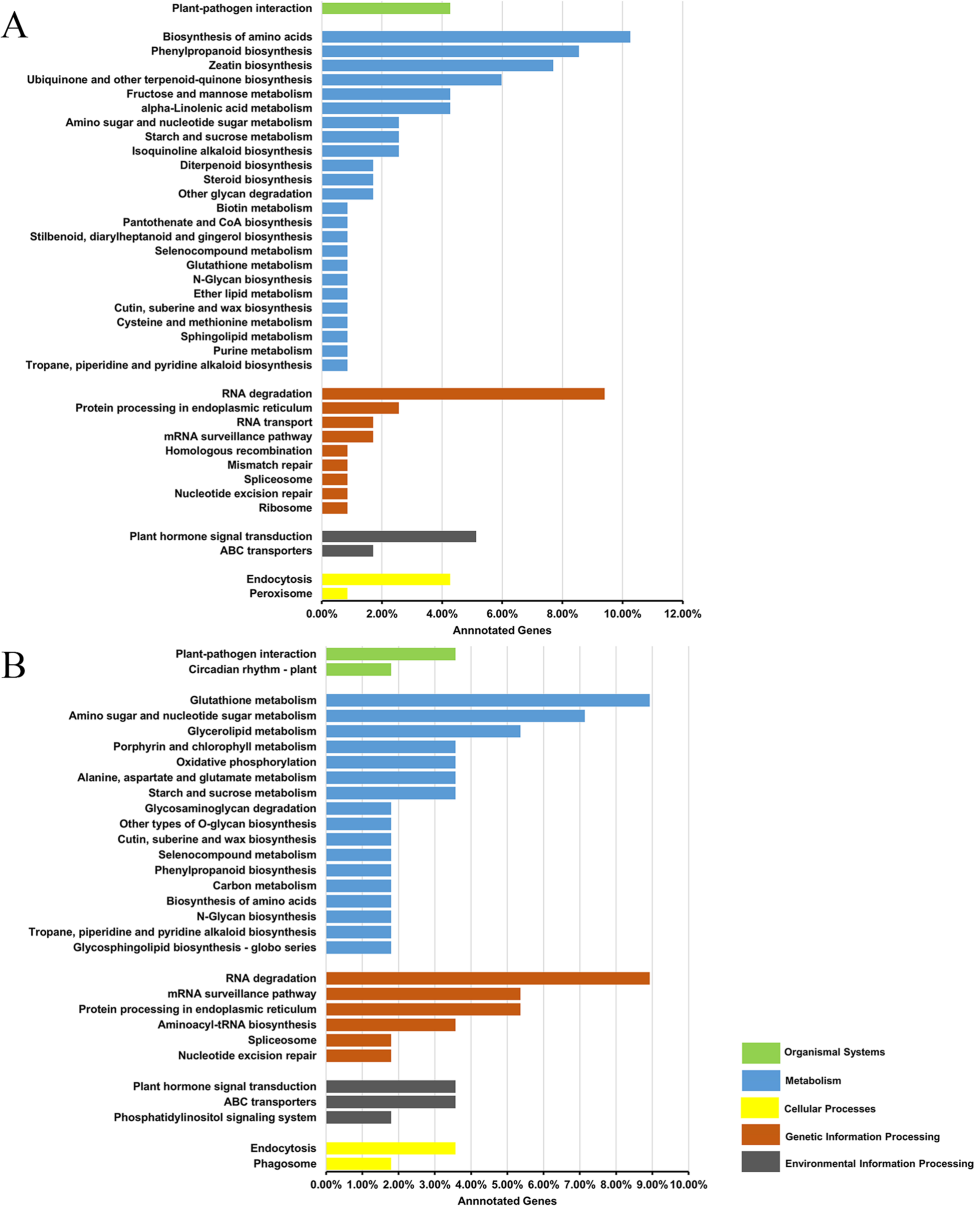
Kyoto encyclopedia of genes and genomes (KEGG) classification map of the target genes of the differentially expressed miRNAs. **A** KEGG classification map of the target genes of the differentially expressed miRNAs for 7 dpi compared with 0 dpi in resistant line. **B** KEGG classification map of the target genes of the differentially expressed miRNAs for 14 dpi compared with 0 dpi in resistant line. The left y-axis shows different KEGG metabolic pathways, and the x-axis represents the number of genes annotated to the pathway and their proportion to the total number of genes annotated

According to Kyoto Encyclopedia of Genes and Genomes(KEGG)databases [40–42], we found that under the cellular processes, environment information processing, genetic information processing, metabolism, organismal systems categories: (1) From 0 dpi to 7 dpi, the number of genes annotated to RNA degradation (11, 9.40%) and biosynthesis of amino acids (12, 10.26%) accounted for a higher proportion of the total annotated genes in the resistant line. The proportion of genes annotated to endocytosis (124, 5.09%), plant hormone signal transduction (133, 5.46%), and plant-pathogen interaction (132, 5.41%) pathways were higher in the susceptible line (Fig. 6A, Additional file 8: Figure S5A). (2) From 0 to 14 dpi, RNA degradation (5, 8.93%) and glutathione metabolism (5, 8.93%) annotated in the resistant line had a high proportion of genes in the pathway, while endocytosis (124, 5.09%), plant hormone signal transduction (133, 5.46%), biosynthesis of amino acids (109, 4.47%) and plant-pathogen interaction (132, 5.41%) annotated in the susceptible line had a high proportion of genes in the pathway (Fig. 6B, Additional file 8: Figure S5B). The results of the KEGG analysis also showed that the plant resistance response was a complex process. The genes involved in RNA degradation may play an important role in the resistant line, while the genes involved in endocytosis and plant hormone signal transduction may promote susceptibility in the susceptible line.

### Validations of miRNA expressions and targeting NBS-LRR genes

**Fig. 7:**
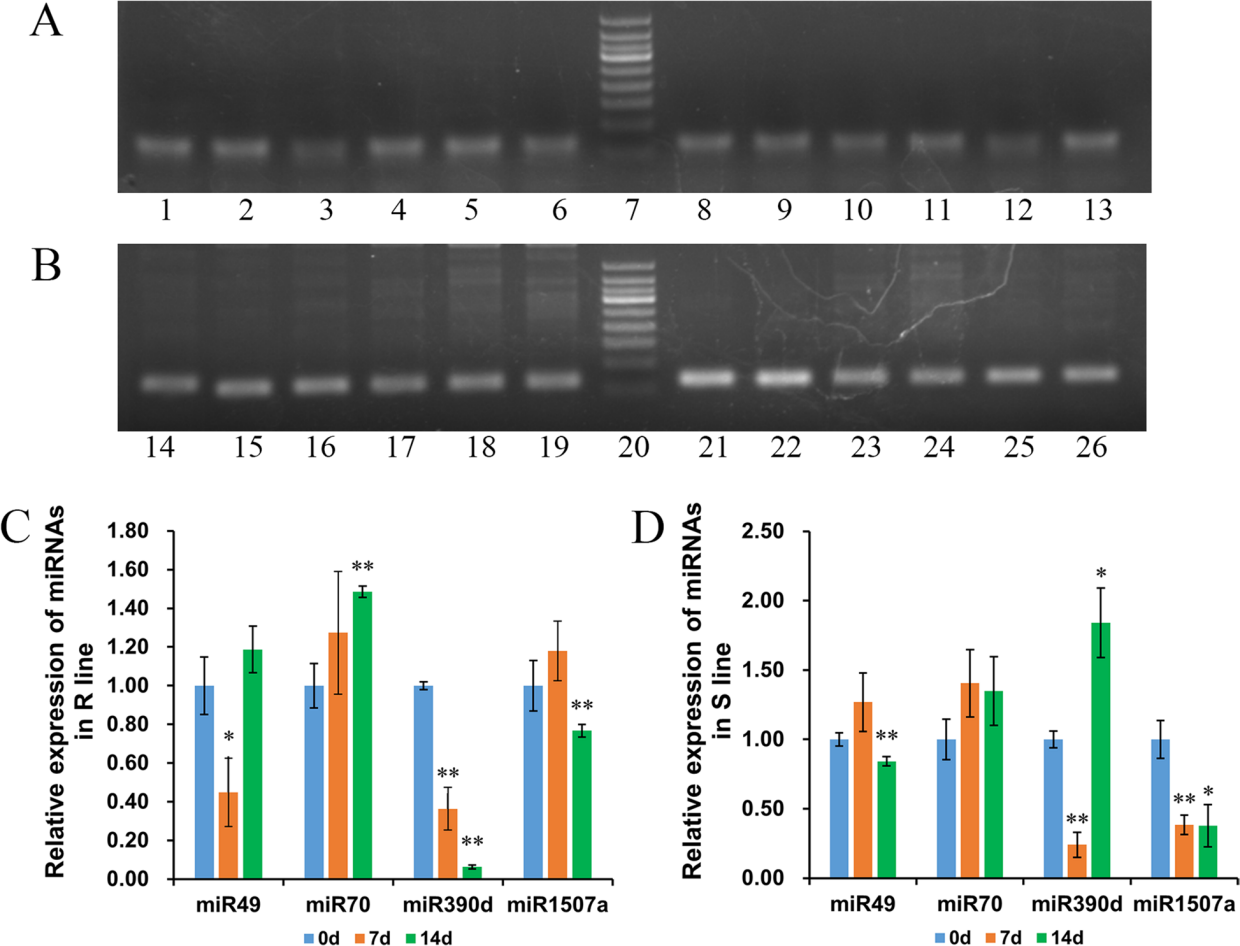
Common RT-PCR and qRT-PCR detection of selected miRNAs. **A** RT-PCR detection of miR49 (1–6 panuels), miR70 (8–13 panuels), miR1507a (14–19 panuels) and miR390d (21–26 panuels) in leaves of two lines inoculated with SC3 at 0 dpi,7 dpi and 14 dpi (The first three bands are from resistant line, the last three bands are from susceptible line in each group). It was cropped from the full-length gels which were presented in Additional file 9: Figure S6. The selected marker was 50 bp DNA ladder (The smallest two bands were 50 bp and 100 bp respectively) and the target band was about 65 bp. **B-C** For the qRT-PCR detection of the above 4 miRNAs in two lines, three biological replicates were set, the selected internal reference gene was U6, and 2^−ΔΔCT^ was used to calculate the expression level of miRNAs

Two known miRNAs (gma-miR1507a and gma-miR390d) and two novel miRNAs (novel-miR49 and novel-miR70), which had contrast expression patterns and may target to NBS-LRR genes were validated by stem-loop RT-PCR and qRT-PCR [43]. The result showed about 65 bp bands at 0 dpi, 7 dpi and 14 dpi in both lines, indicating that these selected miRNAs were real in soybeans (Fig. 7A, the image of the full-length gel was saved as Additional file 9: Figure S6). Then qRT-PCR showed that these four miRNAs’ expression patterns were almost the same as the sequencing results (Fig. 7B-C). For instance, from 0 to 7 dpi, the expression of novel-miR49 was down-regulated in the resistant line but not significantly changed in the susceptible line. The novel-miR70 did not show significant changes in the resistant line.

The prediction maps of folded precursor structure and the target sites of the 2 novel miRNAs were shown in the Additional file 10: Figure S7. We validated the miRNA-target gene interaction by agrobacterium-mediated transient expression in *Nicotiana benthamiana*. The results showed that the expression levels of the 2 target genes decreased to varying degrees compared with the control at the transcriptional level (Additional file 11: Figure S8), indicating that the selected miRNAs may target to the genes and affect gene expression.

## Discussion

In the present study, we present a detailed snapshot of the miRNA expression pattern in host soybean after SMV infection that contributes to a better understanding of the interaction among soybean and SMV and the mechanisms of SMV resistance mediated by miRNAs. We performed deep sequencing and compared the miRNA expression between the resistant and susceptible lines following virus infection at different time intervals.

### Characteristics of miRNAs

In our study, the maximum length of all known miRNAs and novel miRNAs was 21 nt, followed by 24 nt, which was consistent with the length characteristics of miRNAs in plants, including soybean. MiRNAs with different lengths may have different regulatory effects on plants. For instance, 24 nt miRNAs prefer to regulate DNA methylation by binding to AGO4 proteins and ultimately play an important role in the transcriptional silencing of transposons and centromeres [44, 45]. At the same time, miRNAs with 21 and 22 nt in length are more likely to bind to AGO and cleave target genes by forming silencing complexes [46]. Here, we screened many miRNAs that are differentially expressed in two lines and may target the NBS-LRR genes. Many of them are 21 nt in length, including gma-miR2118a-3p, gma-miR390d, gma-miR5041-5p, gma-miR10440, etc.

### The differentially expressed miRNAs may affect the response of soybean to SMV

It is of great significance to study miRNAs with different expression trends in response to SMV infection in both lines. With the help of Venn diagrams, numerous corresponding miRNAs were obtained. They target many different types of genes with many functions, but because of their divergent expression trends, which may make the target genes have various degrees of regulation, and therefore may make plants have different responses to SMV. Plants are often attacked by a variety of pathogens during their growth and development. In the course of long-term evolution, they have acquired a series of complete and complex defense mechanisms [47, 48]. The emergence of resistance genes is one of the reasons [49]. Among them, NBS-LRR (nucleotide binding site-leucine rich repeat) resistance genes accounts for about 80%. A number of NBS-LRR resistance genes have been cloned in Arabidopsis thaliana, rice, tomato, potato and other plants, and they have been found to mediate resistance to *Hyaloperonospora parasitica*, *Magnaporthe oryzae*, *Pseudomonas syringae*, *Phytophthora infestans*. etc. [50–55].

There are many NBS-LRR genes in soybean, but their functions are not well studied at present. By predicting the target genes of differentially expressed miRNAs, we found many miRNAs that may target NBS-LRR resistance genes. For example, the expression level of novel-miR49 at 7 dpi in resistant line was lower than that at 0 dpi, while there was no significant indigenous change in susceptible line within the same time range. *Glyma. 18G287100* belongs to NBS-LRR resistance gene according to gene annotation, and it may be a target gene of novel-miR49; *Glyma.13G187900* may belong to a target gene of gma-miR2118a-3p, and the expression of gma-miR2118a-3p at 7 dpi and 14 dpi in susceptible line was significantly higher than that at 0 dpi, while there was no significant change in the same time point in resistant line. These results implied that down- or up-regulated miRNAs may play negative or positive roles on soybean response to SMV infection.

Previous studies have found that many miRNAs, such as miR160, miR393, miR1510, miR1507a, miR1507c and miR482a, miR168, were actively involved in the response to SMV infection to further affect the expression of downstream target genes through up- or down-regulation, thereby affecting the phenotype of plants [35, 36, 38]. Among them, miR1507a, miR1507c, and miR482a have been confirmed to target NBS-LRR resistance genes [38]. In our study, similar conclusions were also obtained and some novel miRNAs, like novel-miR49 and novel-miR70, were predicted which may also target NBS-LRR resistance genes.

### Multiple pathways may be involved in soybean response to SMV infection

Predicting the target genes of miRNAs would be critical to better understanding the biological functions of these miRNAs in response to SMV infection. In our study, 1020 known and novel miRNAs predicted 20,925 target genes. Of these, 20,913 genes annotation information was obtained. Further, the differentially expressed miRNA target genes were classified by Gene Ontology (GO) analysis. It revealed that from 0 to 7 dpi, the target genes corresponding to the differentially expressed miRNAs in the two lines were highly enriched in terms such as metabolic process, cellular process, cell part, catalytic activity, and binding, and the enrichment of the two lines from 0 to 14 dpi is similar to that from 0 to 7 dpi. In addition, we noticed that with the change of time, the number of genes enriched in certain pathways had changed significantly. For instance, the degree of enrichment of resistant line in terms of detoxification, biological adhesion, etc., has been considerably reduced. The results indicate that these pathways, which are enriched differently at different time points, may play an important role in the process of SMV infecting soybeans.

To further analyse the specific biological functions of miRNAs under the infection of SMV, we performed KEGG analysis on the target genes of differentially expressed miRNAs from the both lines. For instance, from 0 to 7 dpi, the proportion of genes annotated to RNA degradation, biosynthesis of amino acids, and other pathways in resistant line are relatively high, while those in susceptible line are mainly annotated to pathways such as endocytosis, plant hormone signal transduction, and plant-pathogen interaction.

### Identification of candidate genes for disease resistance by miRNA-seq combined with previous mapping results

Soybean contains many NBS-LRR genes, but limited studies were only conducted to describe the role of their involvement in resistance against pathogens. Previous studies showed several SMV resistance genes, including *Rsv*_*1,*_* R*_*SC3Q*_, *R*_*SC11*_, *R*_*SC14Q,*_ and *R*_*SC20*_ were located at 4.295 Mb genomic region between 27 656 895–31 951 960 bp on chromosome 13, and this genomic region is rich in NBS-LRR genes [56–61]. Yuan et al. [62] combined the mapping and genome re-sequencing results, found that *Glyma.13G26380* may be involved in the process of soybean SC3 resistance. Wu et al. [63] mapped the bean common mosaic virus (BCMV) resistance genes in the 58.1 kb interval between BARSOYSSR _13 _ 1114 and SNP-49 through positional cloning, including a CC-NBS-LRR type of resistance gene *Glyma.13G184800*, while *Rsv1-h* responsible to SMV was mapped to almost the same region as the previous SMV resistance allele, indicating that this gene may respond to both viruses. Zheng et al. [59] found three genes including *Glyma.13G25730*, *Glyma.13G*25750 (*Glyma.13G187900*), and *Glyma.13G25950* (*Glyma.13G190300*) were up-regulated in resistant line and down-regulated in susceptible line after SMV infection, while two genes, *Glyma.13G25970* (*Glyma.13G190400*) and *Glyma.13G*26000 (*Glyma.13G190800*) were only expressed in the resistant line. In another study, Li et al. [60] showed that the SC3 resistance might be attributed by several candidate genes such as *Glyma.13G25920* (*Glyma.13G190000*), *Glyma.13G25950*, *Glyma.13G25970,* and *Glyma.13G26000*. Here, we predicted the miRNAs potential target genes may be related to disease-resistant (*i.e., Glyma.13G184800, Glyma.13G187900, Glyma.13G188300, Glyma.13G190300, Glyma.13G190400, Glyma.13G190800, Glyma.13G193100, and Glyma.13G193300*), and they were present in the same genomic region on chromosome 13. Meanwhile, experiments on two novel miRNAs predicted to target NBS-LRR resistance genes showed their authenticity, and the corresponding target genes may also affect the disease resistance response of soybean.

## Conclusions

In the present study, miRNAs regulated by SMV in soybean were identified and characterized by deep sequencing analysis, and some miRNAs related to plant defense mechanisms were found to be responded after virus infection. The function of these miRNAs in soybean resistance to SMV infection may need supplementary study. Even so, our finding helps to advance the knowledge of SMV infection in host plants while also offering new sight for the development of management strategies for SMV infection in the future.

## Methods

### Plant genetic materials and virus source

All soybean materials used in this study are from the National Center for Soybean Improvement (NCSI), Nanjing, China. Our study complies with relevant institutional, national, and international guidelines and legislation. In a former study, we developed a set of NILs from the cross between Nannong1138-2 and Qihuang No.1 using the heterogeneous inbred family method for the *R*_*SC3Q*_ on chromosome 13 of soybean. Of these, two pairs of NILs with significantly contrasting responses to SMV (Resistant (R) and susceptible (S) lines) were used in this study. Formal confirmation of the plant materials has been done by phenotyping and genotyping. An SMV isolate belongs to the strain of SC3, also obtained from the NCSI, was used in this study.

### Virus inoculation and tissue sampling

The inoculum of the virus was prepared from the leaves of the susceptible cultivar, Nannong1138-2. The inoculum preparation, inoculation, and phenotype evaluation were done following the method of Li et al. [7]. The resistant and susceptible lines were grown with three biological replicates in pots and kept in a growth chamber set at 28 °C with 60–70% relative humidity for a photoperiod of 16 /8 h (Light/ Dark). After 10 days, when unifoliate leaves developed, resistant and susceptible lines were inoculated with SC3. The leaf samples were collected at 0 dpi, 7 dpi and 14 dpi, immediately frozen in liquid nitrogen and kept at − 80° C until processed.

### Small RNA libraries preparation and deep sequencing

Total RNA was extracted from soybean leaves by miRcute Plant miRNA Isolation Kit (Tiangen, China). The concentration and integrity of RNA were detected by nanodrop (Thermo Fisher, USA) and Agilent 2100 (Agilent Technologies, USA). Then the sRNA libraries were constructed by using NEB Next Multiplex Small RNA Library Prep Set for Illumina ® (Set 1) (New England Biolabs, USA), including reverse transcription and PCR enrichment after adding adapters to 5’and 3’ of RNA. The PCR products were purified by VAHTSTM DNA Clean Beads (Vazyme, China), and then PAGE gel electrophoresis was performed, and the target fragment was selected for gel cutting and recycling. After confirming the quality and quantity of cDNA libraries, they were sequenced with the single-end read length of 50 bp using an Illumina HiSeq X-ten platform.

### Small RNA analysis and miRNAs prediction

Raw sequencing reads were filtered to ensure the quality of the analysis by follows: (1) discard the low-quality reads, (2) discard the reads without 3’ and 5’ adaptor, (3) discard the reads with unknown base N (N specifies unidentified base) content that was larger than 10%, (4) discard sequences shorter than 18 nt or longer than 30 nt for further analysis. The fragments of the rRNA, tRNA, snRNA, snoRNA, other ncRNA, and repetitive sequences were discarded by bowtie with the support of the Silva database, GtRNAdb database, Rfam database, and Repbase database, and unannotated reads were acquired, together with miRNAs. Further, the unannotated reads were mapped to the soybean reference genome (Wm82.a2.v1). Then, individually mapped reads were run through the miRDeep2 module to quantify known miRNAs (miRBaseV22) and discover novel miRNAs. The novel miRNAs were predicted following the criteria used by the Bayesian model.

### Quantification and differential expression analysis of miRNAs

The expression level of miRNA was normalized as transcripts per million (TPM). The formula is: TPM = read count*1,000,000/mapped reads. DESeq2 was used to detect the differentially expressed miRNAs among the groups. To determine the significance of each miRNA expression difference, we used the value of |log2 fold change (FC)|≥ 1.00 and false discovery rate (FDR) ≤ 0.05 as the criterion. FC shows the ratio of expression levels between the two samples, and FDR is an indicator for differentially expressed miRNAs.

### Prediction of miRNA-targeted genes and gene function analyses

Potential miRNA-targeted genes were predicted using the TargetFinder program by providing the sequences of all miRNAs. The functional annotation of the miRNA-targeted genes to GO terms was performed according to the Gene Ontology database (http://www.geneontology.org/). The obtained results were divided into three groups: biological process, molecular functions, and cellular components. Additionally, these annotated miRNA-targeted genes were further subjected to Kyoto Encyclopedia of Genes and Genomes (KEGG) (http://www.genome.jp/kegg/) pathway enrichment analysis using KOBAS.

### Expression analysis of the miRNA using stem-loop RT-PCR and qRT-PCR

The total RNA was isolated using TRIzol Reagent (Invitrogen, USA) following the user guidelines. For miRNAs, the cDNA was synthesized by RT using the miRNA 1st Strand cDNA Synthesis Kit (by stem-loop) (Vazyme, China) with a special stem-loop RT primer (Additional file 12: Table S4). The authenticity of miRNAs was verified by RT-PCR using Ex Taq (Takara, Japan), PCR procedure: Pre-denatured at 95 °C for 1 min; Reaction at 98 °C for 10 s, 57 °C for 15 s, 72 °C for 20 s, a total of 40 cycles. The cDNA was amplified by real-time RT-PCR using the SYBR green supermix (Vazyme, China) at 95 °C for 5 min, followed by 40 cycles of 95 °C for 15 s, 60 °C for 60 s, and 95 °C for 15 s. The experiment included three biological replicates, and normalized expression levels were measured using the relative quantification (2^−ΔΔCt^) method [64], and data were compared with U6 as the internal reference control.

### Construction of transient expression vectors and agroinfiltration on *N.benthamiana*

The target miRNA and its complementary sequence were used to replace miR319 and miR319* on the pRS300 vector skeleton by overlapping PCR and then ligated between the *Nco* I and *Pml* I restriction sites following the 35S promoter of the pCAMBIA3301 vector using ClonExpress II One Step Cloning Kit (Vazyme, China). The ligation products were transformed into *E. coli* by heat shock method and verified by sequencing. The growth conditions of *N.benthamiana* were: 24 °C temperature, 14 /10 h (day/night) photoperiod. Suspension of *A. tumefaciens EHA105* containing transient expression vector with infiltration solution (150 mM acetosyringone,10 mM MES [pH 5.6] and 10 mM MgCl_2_) to appropriate concentration. The solution was inoculated for about 2 h while the OD = 0.8, and the back of the blade was infiltrated with a 1 ml syringe.

## Supplementary Information

**Additional file 1: Figure S1.** Distribution maps of sRNA types in each library.

**Additional file 2: Table S1.** Summary of read details from resistant and susceptible lines.

**Additional file 3: Figure S2.** The position coverage depth distribution map of reads on the reference genome.

**Additional file 4: Table S2.** Classification of miRNAs family details.

**Additional file 5: Figure S3.** Differential expression of miRNA plot between different groups.

**Additional file 6: Table S3.** Summary of annotated differentially expressed miRNA target genes.

**Additional file 7: Figure S4.** Gene ontology (Go) analysis of differentially expressed miRNAs target genes.

**Additional file 8: Figure S5.** Kyoto encyclopedia of genes and genomes (KEGG) classification map of the differentially expressed miRNAs target genes.

**Additional file 9: Figure S6.** Full length gel for RT-PCR detection of selected 4 miRNAs.

**Additional file 10: Figure S7. **The prediction maps of folded precursor structure (A,the left one is novel-miR49 and the right one is novel-miR70) and the target sites (B) of the 2 novel miRNA.

**Additional file 11: Figure S8.** Relative expression levels of two potential target genes. (A) was for Glyma.02g023800 and (B) was for Glyma.18g287100.Three biological replicates were set, the selected internal reference gene was 18s, and 2^-ΔΔCT^ was used to calculate the expression level of the target gene

**Additional file 12: Table S4.** List of primers used for all the experiments.

## Data Availability

The small RNA raw sequencing data have been deposited in the Sequence Read Archive (SRA) at the National Center for Biotechnology Information (NCBI), and the Bio project number is PRJNA743161(https://www.ncbi.nlm.nih.gov/bioproject/?term=PRJNA743161).

## Abbreviations

miRNA: MicroRNA
nt: Nucleotide
smv: Soybean mosaic virus
NILs: Near-isogenic lines
dpi: Day post-inoculation
pri-miRNA: Primary miRNA
pre-miRNA: Precursor miRNA
RISC: RNA-induced silencing complex
CP: Coat protein
qRT-PCR: Quantitative real-time PCR
sRNA: Small RNA
rRNA: Ribosomal RNA
tRNA: Transfer RNA
snRNA: Small nuclear RNA
snoRNA: Small nucleolar RNA
ncRNA: Non-coding RNA
GO: Gene Ontology
KEGG: Kyoto Encyclopedia of Genes and Genome
NBS-LRR: Nucleotide-binding site leucine-rich repeat

## References

[1] Hill JH, Whitham SA (2014). Control of Virus Diseases in Soybeans - ScienceDirect. Adv Virus Res.

[2] Li K, Zhi HJ (2016). Advances in Resistance to Soybean Mosaic Virus Disease in Soybean. Soybean Science.

[3] Whitham SA, Qi M, Innes RW, Ma W, Lopes-Caitar V, Hewezi T (2016). Molecular Soybean-Pathogen Interactions. Annu Rev Phytopathol.

[4] Yang QH, Li K, Zhi HJ, Gai JY (2014). Genetic Diversity of Chinese Soybean mosaic virus Strains and Their Relationships with Other Plant Potyviruses Based on P3 Gene Sequences. J Integr Agric.

[5] Bao WH, Yan T, Deng XY, Wuriyanghan H (2020). Synthesis of Full-Length cDNA Infectious Clones of Soybean Mosaic Virus and Functional Identification of a Key Amino Acid in the Silencing Suppressor Hc-Pro. Viruses.

[6] Jayaram C, Hill JH, Miller WA. Complete nucleotide sequences of two soybean mosaic virus strains differentiated by response of soybean containing the Rsv resistance gene. J Gen Virol. 1992; 73 ( Pt 8)(8):2067–2077. 10.1099/0022-1317-73-8-2067 1645142

[7] Li K, Yang QH, Zhi HJ, Gai JY (2010). Identification and Distribution of Soybean mosaic virus Strains in Southern China. Plant Dissease.

[8] Wang DG, Tian Z, Li K, Li HW, Huang ZP, Hu GP, Zhang L, Zhi HJ (2013). Identification and variation analysis of soybean mosaic virus strains in Shandong, Henan and Anhui provinces of China. Soybean Science.

[9] Chen XM (2005). MicroRNA biogenesis and function in plants. FEBS Lett.

[10] Jones-Rhoades MW, Bartel DP, Bartel B (2006). MicroRNAS and their regulatory roles in plants. Annu Rev Plant Biol.

[11] Zhang BH, Pan XP, Cobb GP, Anderson TA (2006). Plant microRNA: a small regulatory molecule with big impact. Dev Biol.

[12] Voinnet O (2009). Origin, biogenesis, and activity of plant microRNAs. Cell.

[13] Zhang BH, Wang QL (2015). MicroRNA-based biotechnology for plant improvement. J Cell Physiol.

[14] Yu B, Yang ZY, Li JJ, Minakhina S, Yang MC, Padgett RW, Steward R, Chen XM (2005). Methylation as a Crucial Step in Plant microRNA Biogenesis. Science.

[15] Kurihara Y, Takashi Y, Watanabe Y (2006). The interaction between DCL1 and HYL1 is important for efficient and precise processing of pri-miRNA in plant microRNA biogenesis. RNA.

[16] Lin SL, Chang D, Ying SY (2005). Asymmetry of intronic pre-miRNA structures in functional RISC assembly. Gene.

[17] Nobuta K, McCormick K, Nakano M, Meyers BC (2010). Bioinformatics analysis of small RNAs in plants using next generation sequencing technologies. Methods Mol Biol.

[18] Allen RS, Li JY, Stahle MI, Dubroue A, Gubler F, Millar AA (2007). Genetic analysis reveals functional redundancy and the major target genes of the Arabidopsis miR159 family. Proc Natl Acad Sci.

[19] Curaba J, Talbot M, Li ZY, Helliwell C (2013). Over-expression of microRNA171 affects phase transitions and floral meristem determinancy in barley. BMC Plant Biol.

[20] Zheng ZH, Wang NQ, Jalajakumari M, Blackman L, Shen EH, Verma S, Wang MB, Millar AA (2020). miR159 Represses a Constitutive Pathogen Defense Response in Tobacco. Plant Physiol.

[21] Kang MM, Zhao Q, Zhu DY, Yu JJ (2012). Characterization of microRNAs expression during maize seed development. BMC Genomics.

[22] Peng T, Sun HZ, Qiao MM, Zhao YF, Du YX, Zhang J, Li JZ, Tang GL, Zhao QH (2014). Differentially expressed microRNA cohorts in seed development may contribute to poor grain filling of inferior spikelets in rice. BMC Plant Biol.

[23] Dunoyer P, Lecellier CH, Parizotto EA, Himber C, Voinnet O (2004). Probing the microRNA and small interfering RNA pathways with virus-encoded suppressors of RNA silencing. Plant Cell.

[24] Xia XL, Shao YF, Jiang JF, Du XP, Sheng LP, Chen FD, Fang WM, Guan ZY, Chen SM (2015). MicroRNA Expression Profile during Aphid Feeding in Chrysanthemum (Chrysanthemum morifolium). PLoS One..

[25] Wamiq G, Khan JA (2018). Overexpression of ghr-miR166b generates resistance against Bemisia tabaci infestation in Gossypium hirsutum plants. Planta.

[26] Zhang JY, Xu YY, Huan Q, Chong K (2009). Deep sequencing of Brachypodium small RNAs at the global genome level identifies microRNAs involved in cold stress response. BMC Genomics.

[27] Yang YT, Zhang X, Su YC, Zou JK, Wang ZT, Xu LP, Que YX (2017). miRNA alteration is an important mechanism in sugarcane response to low-temperature environment. BMC Genomics.

[28] Kundu A, Paul S, Dey A, Pal A (2017). High throughput sequencing reveals modulation of microRNAs in Vigna mungo upon Mungbean Yellow Mosaic India Virus inoculation highlighting stress regulation. Plant Sci.

[29] Liang CQ, Liu HW, Hao JJ, Li JQ, Luo LX (2019). Expression profiling and regulatory network of cucumber microRNAs and their putative target genes in response to cucumber green mottle mosaic virus infection. Adv Virol.

[30] Patwa N, Nithin C, Bahadur RP, Basak J (2018). Identification and characterization of differentially expressed Phaseolus vulgaris miRNAs and their targets during mungbean yellow mosaic India virus infection reveals new insight into Phaseolus-MYMIV interaction. Genomics.

[31] Martins TF, Souza PFN, Alves MS, Silva FDA, Arantes MR, Vasconcelos IM, Oliveira JTA (2020). Identification, characterization, and expression analysis of cowpea (Vigna unguiculata [L.] Walp.) miRNAs in response to cowpea severe mosaic virus (CPSMV) challenge. Plant Cell Reports..

[32] Li XY, Wang X, Zhang SP, Liu DW, Duan YX, Dong W (2012). Identification of soybean microRNAs involved in soybean cyst nematode infection by deep sequencing. PLoS One..

[33] Guo N, Ye WW, Wu XL, Shen DY, Wang YC, Xing H, Dou DL (2011). Microarray profiling reveals microRNAs involving soybean resistance to Phytophthora sojae. Genome.

[34] Cui XX, Yan Q, Gan SP, Xue D, Dou DL, Guo N, Xing H (2017). Overexpression of gma-miR1510a/b suppresses the expression of a NB-LRR domain gene and reduces resistance to Phytophthora sojae. Gene.

[35] Yin XC, Wang J, Cheng H, Wang XL, Yu DY (2013). Detection and evolutionary analysis of soybean miRNAs responsive to soybean mosaic virus. Planta.

[36] Chen H, Zhang LR, Yu KF, Wang AM (2015). Pathogenesis of Soybean mosaic virus in soybean carrying Rsv1 gene is associated with miRNA and siRNA pathways, and breakdown of AGO1 homeostasis. Virology.

[37] Chen H, Arsovski AA, Yu KF, Wang AM (2016). Genome-Wide Investigation Using sRNA-Seq, Degradome-Seq and Transcriptome-Seq Reveals Regulatory Networks of microRNAs and Their Target Genes in Soybean during Soybean mosaic virus Infection. PLoS One..

[38] Bao DR, Ganbaatar O, Cui XQ, Yu RN, Bao WH, Falk BW, Wuriyanghan H (2018). Down-regulation of genes coding for core RNAi components and disease resistance proteins via corresponding microRNAs might be correlated with successful Soybean mosaic virus infection in soybean. Mol Plant Pathol.

[39] Friedlander MR, Mackowiak SD, Li N, Chen W, Rajewsky N (2012). miRDeep2 accurately identifies known and hundreds of novel microRNA genes in seven animal clades. Nucleic Acids Res.

[40] Kanehisa M, Goto S (2000). KEGG: Kyoto Encyclopedia of Genes and Genomes. Nucleic Acids Res.

[41] Kanehisa M (2019). Toward understanding the origin and evolution of cellular organisms. Protein Sci.

[42] Kanehisa M, Furumichi M, Sato Y, Ishiguro-Watanabe M, Tanabe M (2021). KEGG: integrating viruses and cellular organisms. Nucleic Acids Res.

[43] Varkonyi-Gasic E, Wu RM, Wood M, Walton EF, Hellens RP (2007). Protocol: a highly sensitive RT-PCR method for detection and quantification of microRNAs. Plant Methods.

[44] Wu L, Zhou H, Zhang Q (2010). DNA Methylation Mediated by a MicroRNA Pathway. Mol Cell.

[45] Wang KT, Su XM, Cui X, Du YC, Zhang SB (2018). Identification and Characterization of microRNA during Bemisia tabaci Infestations in Solanum lycopersicum and Solanum habrochaites. Horticultural Plant Journal.

[46] Brodersen P, Voinnet O (2006). The diversity of RNA silencing pathways in plants. Trends Genet.

[47] Jones DA, Jones JDG (1997). The Role of Leucine-Rich Repeat Proteins in Plant Defences. Adv Bot Res.

[48] Jones DA, Takemoto D (2004). Plant innate immunity - direct and indirect recognition of general and specific pathogen-associated molecules. Curr Opin Immunol.

[49] McHale L, Tan X, Koehl P, Michelmore RW (2006). Plant NBS-LRR proteins: adaptable guards. Genome Biol.

[50] Salmeron JMOG, Rommens CMT, Scofield SR, Kim HSLD, Dahlbeck D, Staskawicz BJ (1996). Tomato Prf is a member of the leucine-rich repeat class of plant disease resistance genes and lies embedded within the Pto kinase gene cluster. Cell.

[51] Botella MAPJ, Frost LN, Bittner-Eddy PD, Beynon JL, Daniels MJ, Holub EB, Jones JD (1998). Three genes of the Arabidopsis RPP1 complex resistance locus recognize distinct Peronospora parasitica avirulence determinants. Plant Cell.

[52] van der Biezen EAFC, Kahn K, Jones JD (2002). Arabidopsis RPP4 is a member of the RPP5 multigene family of TIR-NB-LRR genes and confers downy mildew resistance through multiple signalling components. Plant J.

[53] Huang SW, van der Vossen EA, Kuang H, Vleeshouwers VG, Zhang N, Borm TJ, van Eck HJ, Baker B, Jacobsen E, Visser RG (2005). Comparative genomics enabled the isolation of the R3a late blight resistance gene in potato. Plant J.

[54] Qu S, Liu G, Zhou B, Bellizzi M, Zeng L, Dai L, Han B, Wang GL (2006). The broad-spectrum blast resistance gene Pi9 encodes a nucleotide-binding site-leucine-rich repeat protein and is a member of a multigene family in rice. Genetics.

[55] Liu Y, Liu B, Zhu X, Yang J, Bordeos A, Wang G, Leach JE, Leung H (2013). Fine-mapping and molecular marker development for Pi56(t), a NBS-LRR gene conferring broad-spectrum resistance to Magnaporthe oryzae in rice. Theor Appl Genet.

[56] Shi AN, Chen PY, Li DX, Zheng CM, Hou AF, Zhang B (2008). Genetic confirmation of 2 independent genes for resistance to soybean mosaic virus in J05 soybean using SSR markers. J Hered.

[57] Bai L, Li HC, Ma Y, Wang DG, Liu N, Zhi HJ (2009). Inheritance and gene mapping of resistance to soybean mosaic virus SC-11 in soybean. Soybean Science.

[58] Ma Y, Wang DG, Li HC, Zheng GJ, Yang YQ, Li HW, Zhi HJ (2011). Fine mapping of the *R*_*SC14Q*_ locus for resistance to soybean mosaic virus in soybean. Euphytica.

[59] Zheng GJ, Yang YQ, Ma Y, Yang XF, Chen SY, Ren R, Wang DG, Yang ZL, Zhi HJ (2014). Fine Mapping and Candidate Gene Analysis of Resistance Gene R to Soybean mosaic virus in Qihuang 1. J Integr Agric.

[60] Li C, Adhimoolam K, Yuan Y, Yin JL, Ren R, Yang YQ, Zhi HJ (2017). Identification of candidate genes for resistance to Soybean mosaic virus strain SC3 by using fine mapping and transcriptome analyses. Crop Pasture Sci.

[61] Karthikeyan A, Li K, Li C, Yin JL, Li N, Yang YH, Song YP, Ren R, Zhi HJ, Gai JY (2018). Fine-mapping and identifying candidate genes conferring resistance to Soybean mosaic virus strain SC20 in soybean. Theor Appl Genet.

[62] Yuan Y, Yang YQ, Shen YC, Yu KS, Wang LQ, Ren R, Yin JL, Zhi HJ (2019). Mapping and functional analysis of candidate genes involved in resistance to soybean (Glycine max) mosaic virus strain SC3. Plant Breeding.

[63] Wu M, Wu WP, Liu CC, Liu YN, Wu XY, Ma FF, Zhu AQ, Yang JY, Wang B, Chen JQ (2018). A bean common mosaic virus (BCMV)-resistance gene is fine-mapped to the same region as Rsv1-h in the soybean cultivar Suweon 97. Theor Appl Genet.

[64] Livak KJ, Schmittgen TD (2001). Analysis of relative gene expression data using real-time quantitative PCR and the 2(-Delta Delta C(T)) Method. Methods.

