# Supplementary figures and images for: Discovery and characterization of differentially expressed soybean miRNAs and their targets during soybean mosaic virus infection unveils novel insight into Soybean-SMV interaction
Source: BMC Genomics. 2022 Mar 2;23:171. doi: 10.1186/s12864-022-08385-z (PMC8889786; doi:10.1186/s12864-022-08385-z)

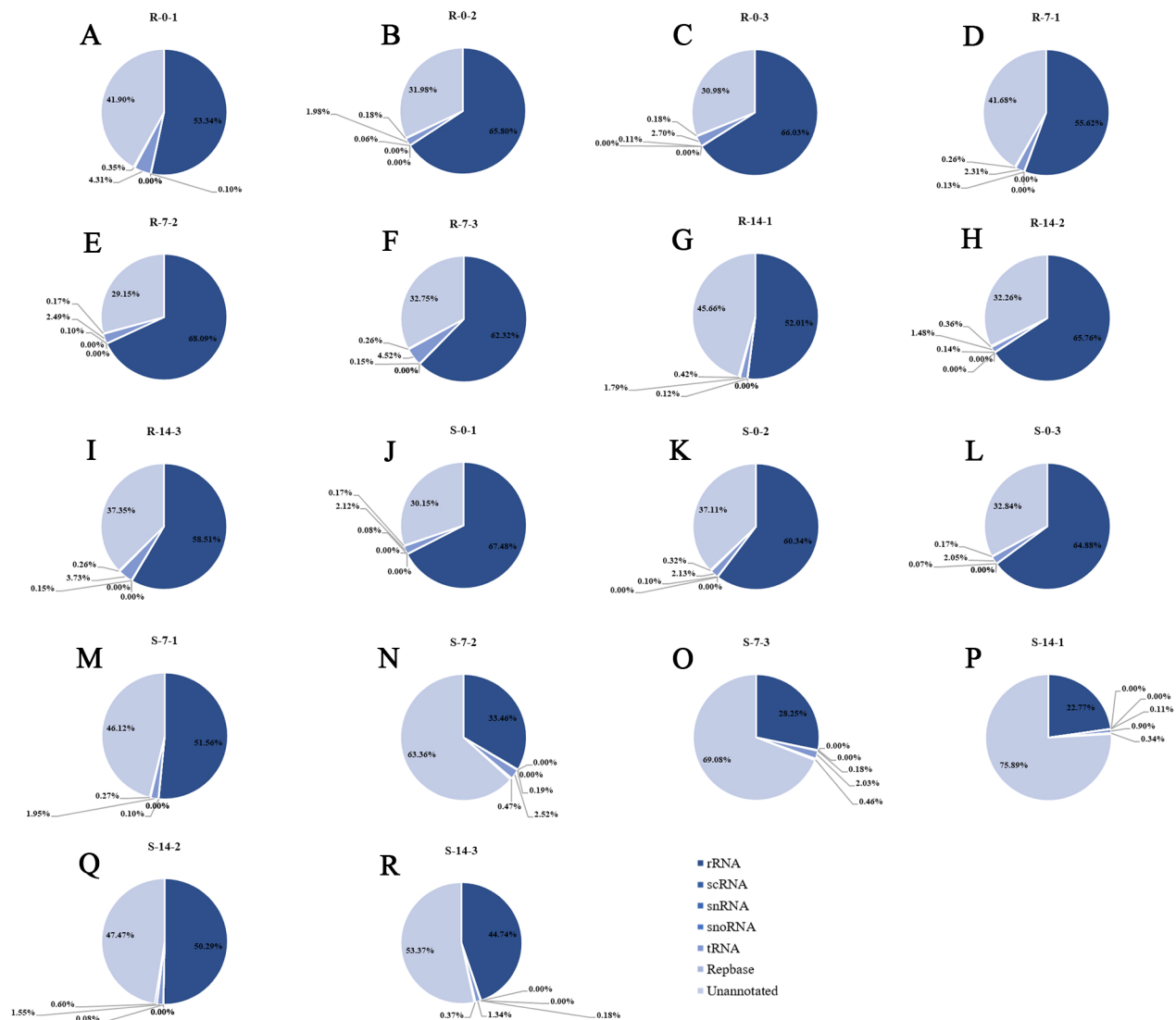

**Figure S1** Distribution maps of sRNA types in each library.

A-R: Percentage of small RNAs in 18 samples.

Supplement: Supplementary file 1 — Additional file 1: Figure S1. Distribution maps of sRNA types in each library. [file 12864_2022_8385_MOESM1_ESM.pdf]
