# Supplementary material for: Discovery and characterization of differentially expressed soybean miRNAs and their targets during soybean mosaic virus infection unveils novel insight into Soybean-SMV interaction
Source: BMC Genomics. 2022 Mar 2;23:171. doi: 10.1186/s12864-022-08385-z (PMC8889786; doi:10.1186/s12864-022-08385-z)
Supplement: Supplementary file 3 — Additional file 3: Figure S2. The position coverage depth distribution map of reads on the reference genome. [file 12864_2022_8385_MOESM3_ESM.pdf]

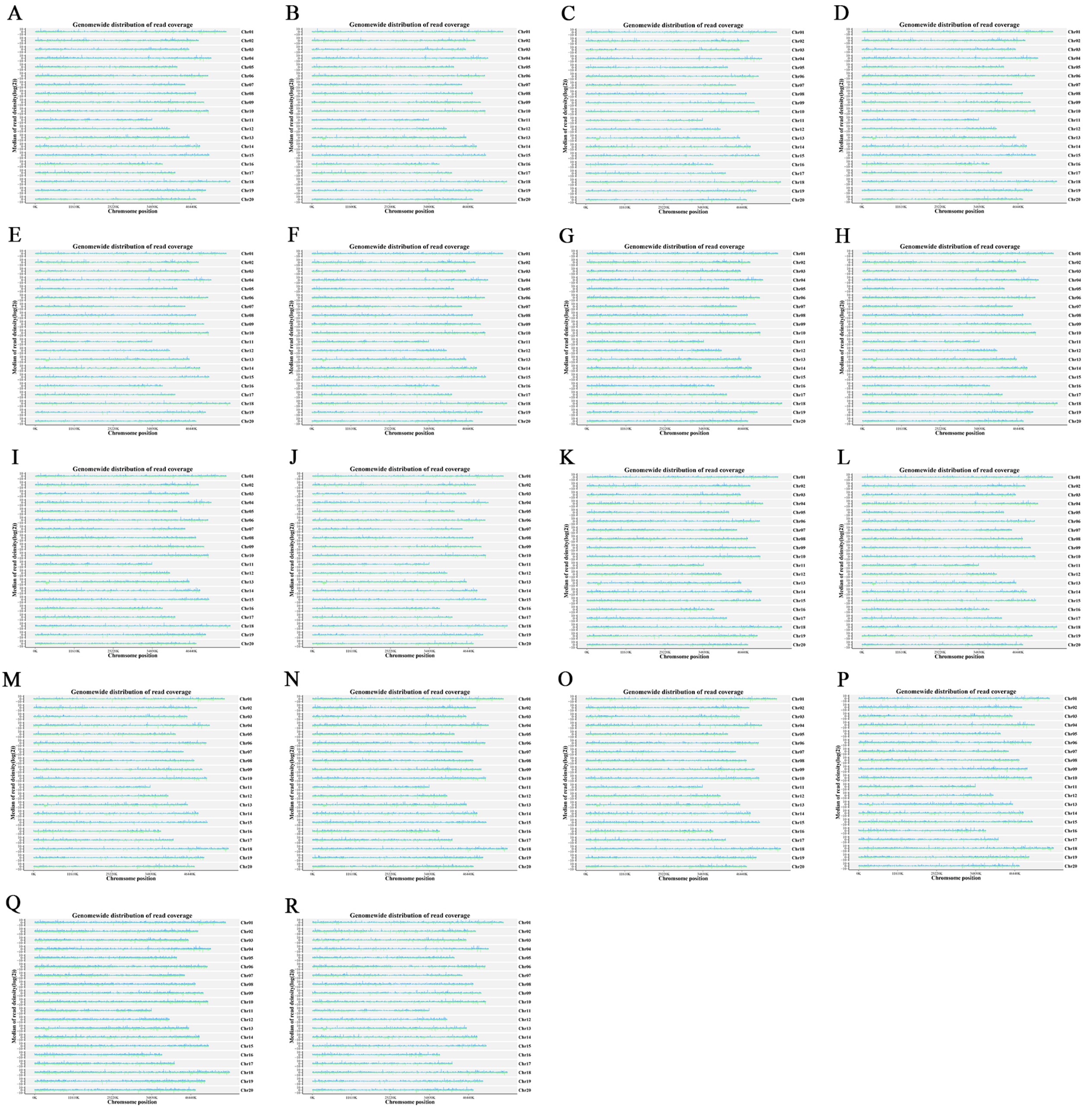

Figure S2 The position and coverage depth distribution map of reads on the reference genome. A-R: Related information in 18 libraries from R-0-1 to S-14-3. The abscissa is the position of the chromosome; the ordinate is the logarithmic value of the coverage depth of the corresponding position on the chromosome with 2 as the base.
