# Supplementary material for: Discovery and characterization of differentially expressed soybean miRNAs and their targets during soybean mosaic virus infection unveils novel insight into Soybean-SMV interaction
Source: BMC Genomics. 2022 Mar 2;23:171. doi: 10.1186/s12864-022-08385-z (PMC8889786; doi:10.1186/s12864-022-08385-z)
Supplement: Supplementary file 5 — Additional file 5: Figure S3. Differential expression of miRNA plot between different groups. [file 12864_2022_8385_MOESM5_ESM.pdf]

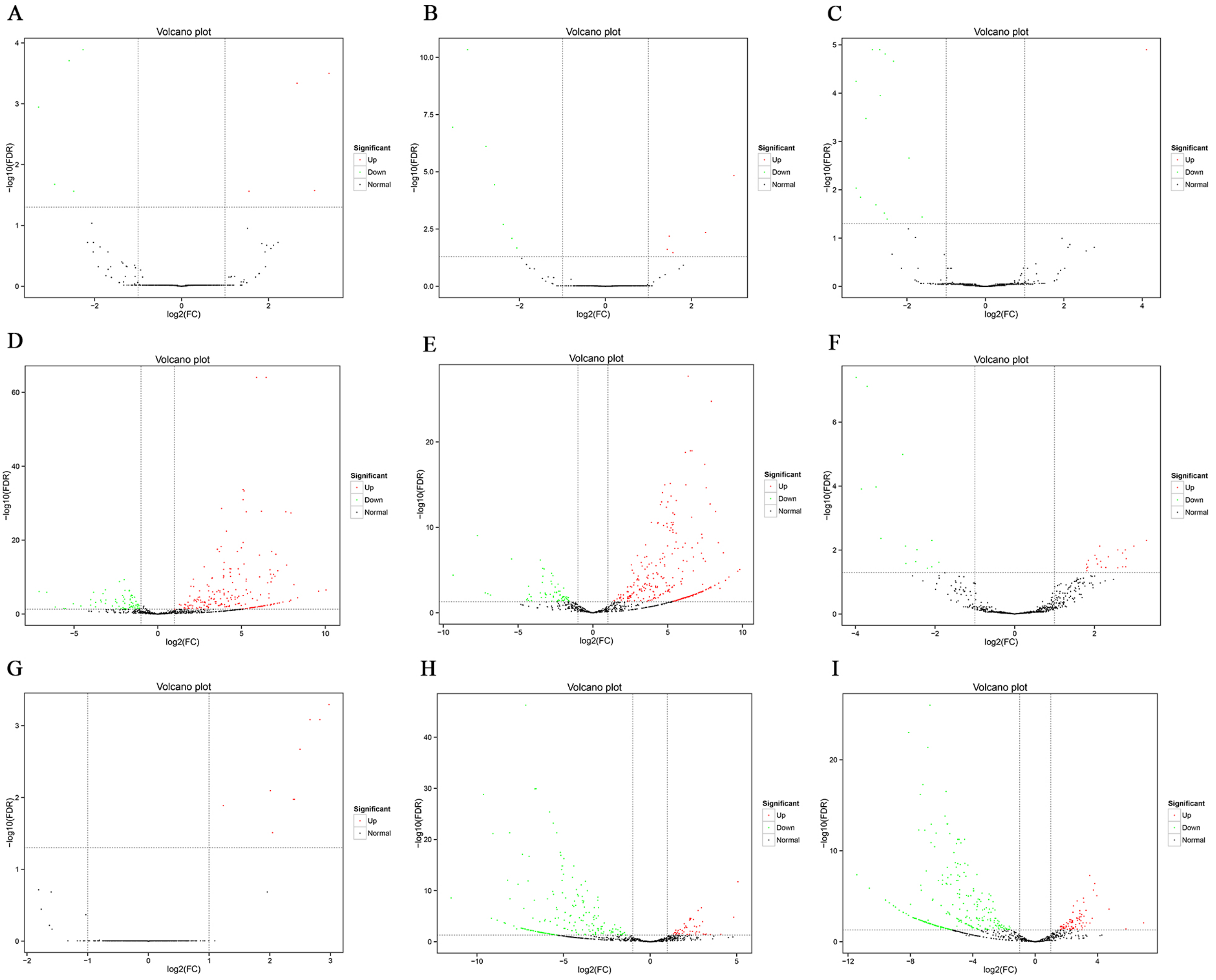

Figure S3 Differential expression of miRNA volcano plot between different groups.

Note:

A: R-0-1, R-0-2, R-0-3 VS R-7-1, R-7-2, R-7-3

B: R-0-1, R-0-2, R-0-3 VS R-14-1, R-14-2, R-14-3

C: R-7-1, R-7-2, R-7-3 VS R-14-1, R-14-2, R-14-3

D: S-0-1, S-0-2, S-0-3 VS S-7-1, S-7-2, S-7-3

E: S-0-1, S-0-2, S-0-3 VS S-14-1, S-14-2, S-14-3

F: S-7-1, S-7-2, S-7-3 VS S-14-1, S-14-2, S-14-3

G: S-0-1, S-0-2, S-0-3 VS R-0-1, R-0-2, R-0-3

H: S-7-1, S-7-2, S-7-3 VS R-7-1, R-7-2, R-7-3

I:S-14-1, S-14-2, S-14-3 VS R-14-1, R-14-2, R-14-3

Each point in the volcano map represents a miRNA, and the abscissa represents the logarithm of the fold difference in the expression of a miRNA between the two samples, the ordinate represents the negative logarithm of the difference error rate. The Blue dots represent undifferentially expressed miRNAs, red dots represent up-regulated miRNAs, and green dots represent down-regulated miRNAs.
