# Supplementary material for: Discovery and characterization of differentially expressed soybean miRNAs and their targets during soybean mosaic virus infection unveils novel insight into Soybean-SMV interaction
Source: BMC Genomics. 2022 Mar 2;23:171. doi: 10.1186/s12864-022-08385-z (PMC8889786; doi:10.1186/s12864-022-08385-z)
Supplement: Supplementary file 8 — Additional file 8: Figure S5. Kyoto encyclopedia of genes and genomes (KEGG) classification map of the differentially expressed miRNAs target genes. [file 12864_2022_8385_MOESM8_ESM.pdf]

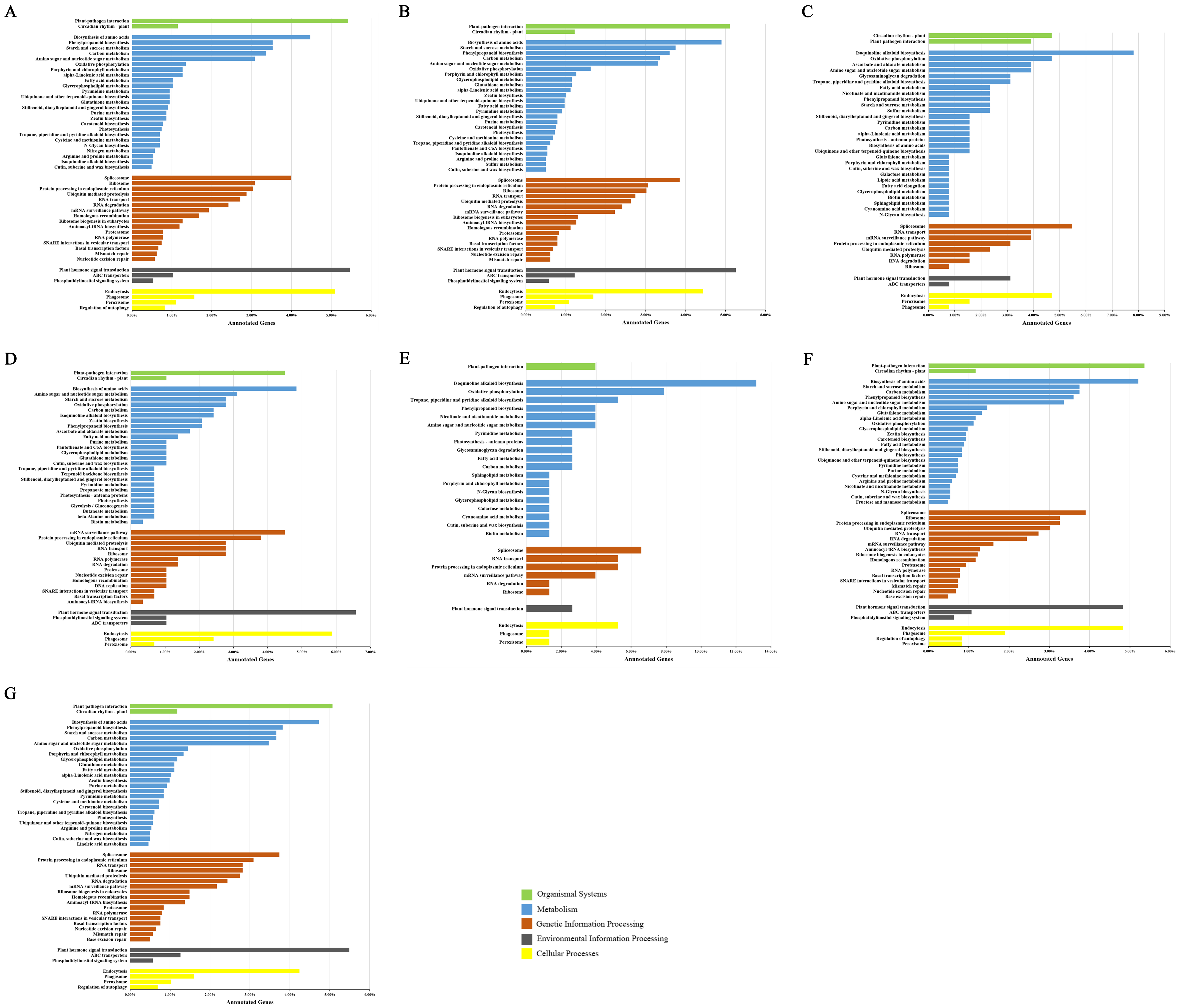

Figure S5 Kyoto encyclopedia of genes and genomes (KEGG) classification map of the differentially expressed miRNAs target genes.

Note

A: S-0-1, S-0-2, S-0-3 VS S-7-1, S-7-2, S-7-3. KEGG classification map of the target genes of the differentially expressed miRNAs for 7 dpi compared with 0 dpi in susceptible line. The following are similar.

B: S-0-1, S-0-2, S-0-3 VS S-14-1, S-14-2, S-14-3

C: R-7-1, R-7-2, R-7-3 VS R-14-1, R-14-2, R-14-3

D: S-7-1, S-7-2, S-7-3 VS S-14-1, S-14-2, S-14-3

E: S-0-1, S-0-2, S-0-3 VS R-0-1, R-0-2, R-0-3

F: S-7-1, S-7-2, S-7-3 VS R-7-1, R-7-2, R-7-3

G:S-14-1, S-14-2, S-14-3 VS R-14-1, R-14-2, R-14-3
