# Supplementary material for: Discovery and characterization of differentially expressed soybean miRNAs and their targets during soybean mosaic virus infection unveils novel insight into Soybean-SMV interaction
Source: BMC Genomics. 2022 Mar 2;23:171. doi: 10.1186/s12864-022-08385-z (PMC8889786; doi:10.1186/s12864-022-08385-z)
Supplement: Supplementary file 9 — Additional file 9: Figure S6. Full length gel for RT-PCR detection of selected 4 miRNAs. [file 12864_2022_8385_MOESM9_ESM.pdf]

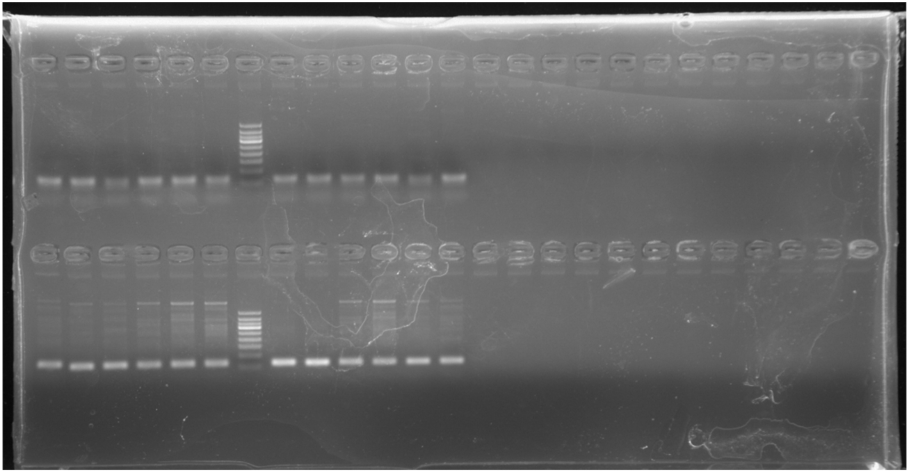

Figure S6 Full-length gel for RT-PCR detection of selected 4 miRNAs.

The upper left is novel-miR49 and the upper right is novel-miR70;The bottom left is gma-miR1507a and the bottom right is gma-miR390d.
