# Supplementary material for: Discovery and characterization of differentially expressed soybean miRNAs and their targets during soybean mosaic virus infection unveils novel insight into Soybean-SMV interaction
Source: BMC Genomics. 2022 Mar 2;23:171. doi: 10.1186/s12864-022-08385-z (PMC8889786; doi:10.1186/s12864-022-08385-z)
Supplement: Supplementary file 10 — Additional file 10: Figure S7. The prediction maps of folded precursor structure (A,the left one is novel-miR49 and the right one is novel-miR70) and the target sites (B) of the 2 novel miRNA. [file 12864_2022_8385_MOESM10_ESM.pdf]

A

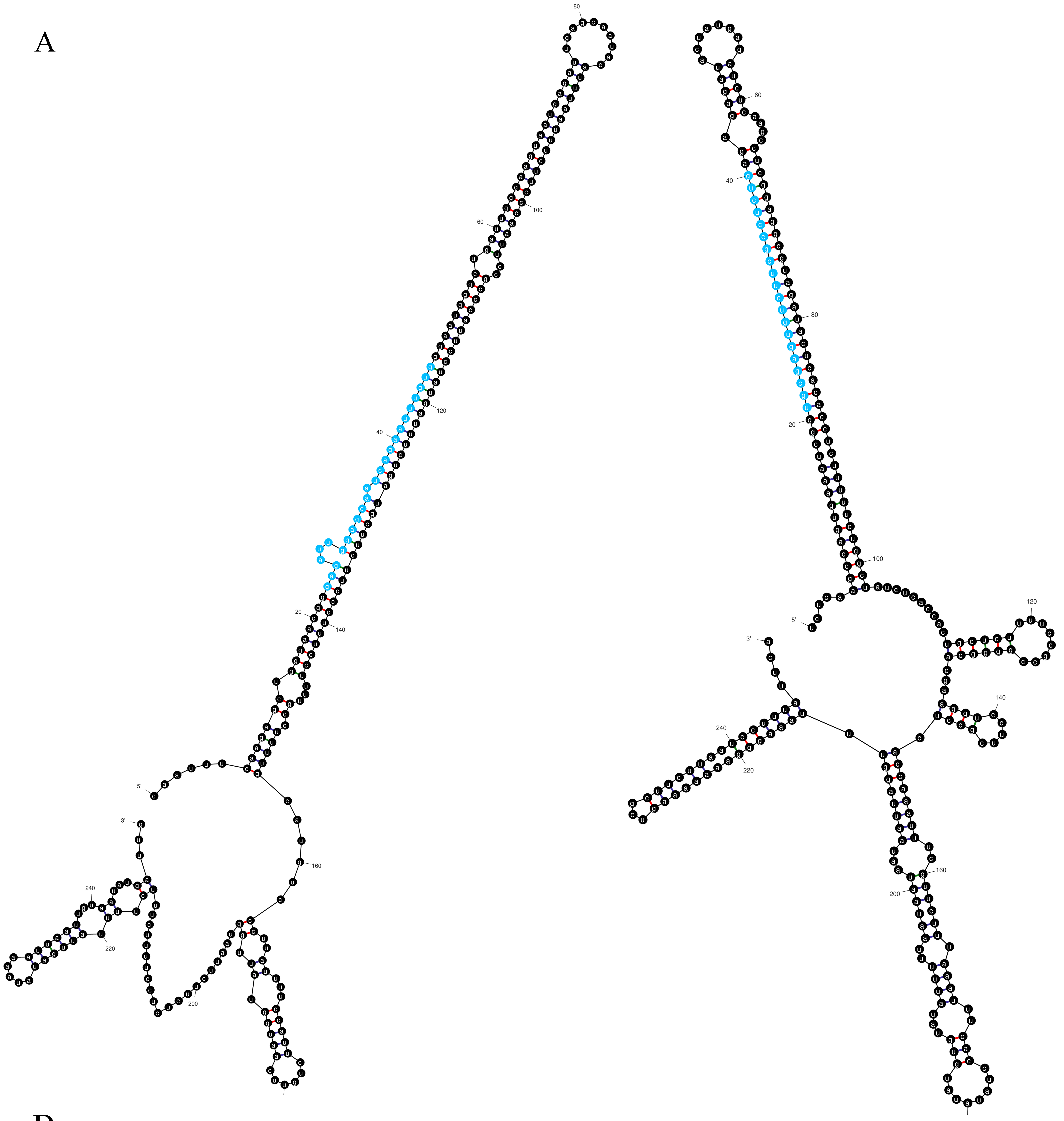

B

```
Glyma. 18G287100  2250  C A C  A A A G U U U G G U U G C U U C A A C U C C  2274
                        | | | | | | ∘ | ∘ | | ∘ | | | | | ∘ | | | ∘ ∘ ∘ |
Novel-miR49         25  G U G U U U A A G A C U  A A C G A G  G U U A G A G   1
```

```
Glyma. 02g023800  63  C A G A G G U G A G G  A C A C A C G C U  82
                      | | | | | | ∘ | | ∘ | | | | | | ∘ | | ∘
Novel-miR70       20  G U C U C C G C U U C U G U G A G C G U  1
```

Figure S7 The prediction maps of folded precursor structure (A,the left one is novel-miR49 and the right one is novel-miR70) and the target sites (B) of the 2 novel miRNAs.
