# Supplementary material for: Discovery and characterization of differentially expressed soybean miRNAs and their targets during soybean mosaic virus infection unveils novel insight into Soybean-SMV interaction
Source: BMC Genomics. 2022 Mar 2;23:171. doi: 10.1186/s12864-022-08385-z (PMC8889786; doi:10.1186/s12864-022-08385-z)
Supplement: Supplementary file 11 — Additional file 11: Figure S8. Relative expression levels of two potential target genes. (A) was for Glyma.02g023800 and (B) was for Glyma.18g287100.Three biological replicates were set, the selected internal reference gene was 18s, and 2-ΔΔCT was used to calculate the expression level of the target gene [file 12864_2022_8385_MOESM11_ESM.pdf]

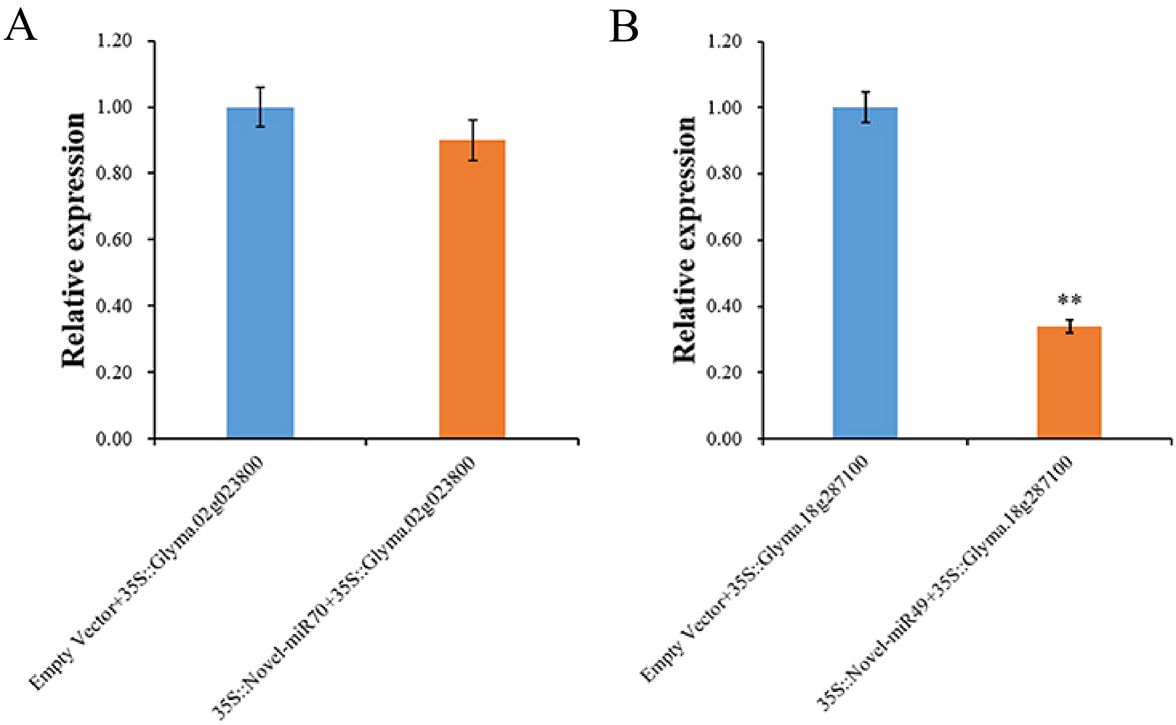

Figure S8 Relative expression levels of two potential target genes. (A) was for Glyma.02g023800 and (B) was for Glyma.18g287100.Three biological replicates were set, the selected internal reference gene was 18s**,** and 2-ΔΔCT was used to calculate the expression level of the target genes.
